# Effect of exercise on brain-derived neurotrophic factors in middle-aged and older adults with type 2 diabetes mellitus: a systematic review and meta-analysis

**DOI:** 10.3389/fphys.2025.1599980

**Published:** 2025-08-26

**Authors:** Zhihua Li, Zhibo Cui, Tong Wang, Haoyu Zheng, Kaixing Li, Chengbo Yang

**Affiliations:** School of Sport and Training, Chengdu Sport University, Chengdu, China

**Keywords:** exercise, brain-derived neurotrophic factors, middle-aged and older adults, type 2 diabetes, meta-analysis

## Abstract

**Background:**

Although previous studies have indicated that exercise can improve brain-derived neurotrophic factor (BDNF) levels in middle-aged and older adults with type 2 diabetes mellitus (T2DM), discrepancies remain among the findings. Therefore, this study aims to determine the impact of exercise on BDNF concentrations in middle-aged and older adults with T2DM.

**Methods:**

A systematic search was conducted across multiple databases, including PubMed, Embase, EBSCO, Cochrane Library, CNKI, and Web of Science, covering the period from the inception of each database to October 2024. The search process adhered to the PRISMA and PERSiST guidelines. Two independent evaluators were responsible for conducting the search, screening results, extracting data, and assessing study quality. A random-effects model was employed to calculate the standardized mean difference (SMD) and 95% confidence interval (CI).

**Results:**

This meta-analysis included 13 studies involving 206 middle-aged and elderly individuals with T2DM. The results showed that exercise effectively increased BDNF levels in middle-aged and elderly individuals with T2DM (SMD = 0.73, 95% CI: 0.07–1.39, *p*< 0.001). Subgroup analysis revealed that aerobic exercise and combined exercise did not significantly increase BDNF levels. Chronic exercise (SMD = 1.04, 95% CI: 0.09 to 1.98, *p*= 0.03) and weekly exercise duration exceeding 150 min (SMD = 1.56, 95% CI: 0.35 to 2.77, *p*= 0.01) significantly increased BDNF levels in middle-aged and older adults with T2DM. In terms of detection methods, non-instant blood sampling (SMD = 1.27, 95% CI: 0.24 to 2.31, *p*= 0.02) and serum BDNF testing (SMD = 0.94, 95% CI: 0.22 to 1.66, *p*= 0.01) were associated with significant increases in BDNF concentrations. There was no significant difference in the effect of diabetes duration ≥10 years *versus*<10 years on BDNF levels in middle-aged and older adults with T2DM.

**Conclusion:**

Exercise has a significant positive effect on BDNF levels in middle-aged and older adults with T2DM. Chronic exercise and exercise lasting more than 150 min per week have a more significant effect on increasing BDNF levels in middle-aged and older adults with T2DM. In addition, when non-immediate blood sampling methods are used to measure serum BDNF levels after exercise, a significant increase in BDNF concentration is observed.

**Systematic Review Registration:**

https://www.crd.york.ac.uk/prospero/#myprospero, identifier CRD42024621098.

## 1 Introduction

Type 2 diabetes mellitus (T2DM) is characterized by insulin resistance in peripheral tissues like skeletal muscle, liver, and fat tissue, which may also affect the brain, coupled with inadequate compensatory insulin release from pancreatic β-cells, culminating in persistent hyperglycemia (Staiger et al., 2009). T2DM represents up to 90% of diabetes cases globally, with the greatest prevalence observed among middle-aged and elderly populations (Ortiz-Martínez et al., 2022). Therefore, it is crucial to prevent and manage T2DM in older adults (Khan et al., 2020).

In the brain, Brain-derived neurotrophic factor (BDNF) is a widely distributed neurotrophic factor that plays a vital role in human metabolic response modulation. It is considered a possible biomarker for forecasting the development of T2DM (Rothman et al., 2012; Colucci-D'Amato et al., 2020; Sharma et al., 2021). A study examining BDNF concentrations among older adults with diabetes in China showed that those with T2DM had significantly lower BDNF levels than healthy individuals, and females had higher levels than males (He and Wang, 2014). This observation may be attributed to the suppression of BDNF secretion in the hippocampus under hyperglycemic conditions (Krabbe et al., 2007). The evidence suggests that diabetes negatively impacts BDNF levels among the elderly. Further studies have shown that BDNF levels are an important predictor of diabetes (Passaro et al., 2015). In older adults with type T2DM, a longer duration of diabetes is linked to lower BDNF levels, indicating a negative correlation (Li et al., 2016). Additionally, studies indicate that a reduction in BDNF levels past a certain limit may negatively affect glucose metabolism, thus raising the risk of diabetes and its associated complications (Moosaie et al., 2023). BDNF also plays a crucial regulatory role in skeletal muscle metabolism (Matthews et al., 2015), boosting glucose use in skeletal muscles and thereby improving the glucose metabolism in patients with T2DM (Yamanaka et al., 2007). Therefore, increasing BDNF levels is of critical importance for the prevention of diabetes.

Exercise is acknowledged as a powerful approach for enhancing diabetes management, boosting overall health, and greatly lowering the risk of complications related to diabetes (Kanaley et al., 2022). Research indicates that a suitable exercise regimen can enhance body composition, regulate blood glucose levels, and influence BDNF expression in individuals with T2DM (Donyaei et al., 2024). A multitude of studies have explored how exercise impacts BDNF levels. Studies on animals have demonstrated that both aerobic and resistance exercises can elevate BDNF levels in rodents and enhance BDNF expression in the brain (Shekarchian et al., 2023; Wang et al., 2023; King et al., 2024). However, most studies have primarily concentrated on rodent models, illustrating the effects of exercise in these animals. Although studies are ongoing, there is still a lack of conclusive proof about how exercise impacts BDNF levels in humans. Recently, there has been an increase in research examining how exercise affects BDNF levels in middle-aged and older adults with T2DM. These investigations reveal that exercise can significantly enhance BDNF levels in this population (Brinkmann et al., 2017; Luo et al., 2022; Donyaei et al., 2024). However, it is crucial to acknowledge that results are not always consistent, as some research has shown no notable rise in BDNF levels after exercise in older adults with T2DM (Swift et al., 2012; Ghodrati et al., 2023; Silveira-Rodrigues et al., 2023a; Silveira-Rodrigues et al., 2023b).

Given the increasing number of trials investigating the effects of exercise on BDNF levels in middle-aged and older adults with T2DM, the results remain inconsistent, potentially due to differences in exercise protocols. The aim of this systematic review and meta-analysis is to determine the impact of physical exercise on BDNF levels in middle-aged and older adults with T2DM and to identify the optimal physical exercise intervention for enhancing BDNF levels in this population.

## 2 Methods

The systematic review and meta-analysis adhered strictly to the PRISMA statement and PERSiST guidelines (Page et al., 2021; Ardern et al., 2022). This study’s protocol is registered with the International Prospective Register of Systematic Reviews (PROSPERO) and has the registration number (CRD42024621098).

### 2.1 Information sources and search strategy

A thorough search of the literature was carried out across several databases, including PubMed, CNKI, Embase, Web of Science, EBSCO, and Cochrane Library, including publications from the establishment of each database through November 2024, without any language restrictions on the documents obtained. The search strategy entailed a manual review of existing literature employing the following keywords: exercise, physical activity, Type 2 diabetes mellitus, brain-derived neurotrophic factor, and neurotrophic factor. A thorough search strategy is provided in Supplementary Material S1. Two authors (ZL and ZC), independently reviewed the literature, assessing the title, abstract, and full text of each article to decide if they met the inclusion criteria.

### 2.2 Inclusion criteria

To qualify for inclusion in this study, trials must meet the following criteria, derived from the PICO framework (da Costa Santos et al., 2007):

1. Participants: T2DM middle-aged and older adults with a mean age ≥ 45 (Zhang and Ni, 2023). Studies involving individuals with impaired glucose tolerance, insulin resistance, or prediabetes are excluded. There are no restrictions regarding the participants’ gender, ethnicity, or nationality.
2. Intervention: Any form of exercise.
3. Comparison: BDNF levels post-exercise will be compared to BDNF levels pre-exercise.
4. Outcome: Levels of BDNF.
5. Study Design: Both randomized and non-randomized types of controlled trials.

### 2.3 Exclusion criteria

The following studies will be excluded from this review: those lacking an exercise intervention, individuals with non-diabetes-related diseases, professional athletes or individuals with a history of regular physical exercise, studies involving non-exercise interventions, cross-sectional studies, review articles, correlation studies, qualitative research, case reports, experimental protocols, theses, and studies with incomplete data or registration information. No gender-based criteria were used for inclusion or exclusion in the systematic review and meta-analysis.

### 2.4 Quality assessment

The studies included were evaluated for potential bias using the Cochrane Risk of Bias Tool (Cumpston et al., 2019). Two researchers (ZL and ZC) independently conducted quality assessments of each article using this tool. When disagreements arose during the evaluation, a third researcher (HZ) participated in discussions to resolve differences, ensuring the results were objective and accurate. The Cochrane Risk of Bias Tool evaluates multiple areas of bias, including: random sequence generation, allocation concealment, blinding of participants and personnel, blinding of outcome assessors, incomplete outcome data, selective outcome reporting, and other sources of bias. Each domain is evaluated and labeled as having a low, high, or uncertain risk of bias (Savović et al., 2012).

### 2.5 Screening process and data collection

In selecting studies and extracting data, we adhered to the PRISMA statement guidelines (Moher et al., 2009). Articles retrieved from the databases were consolidated into a single database, and duplicate records were removed using EndNote 20 software (Clarivate, EndNote Team, Pennsylvania, USA). The screening was independently carried out by researchers (ZL and ZC), with any disagreements being resolved through consultation with a third researcher (HZ). After reviewing the titles and abstracts, studies that clearly did not align with the inclusion criteria were initially excluded. The articles that remained were then fully reviewed to decide if they were eligible for the study. Furthermore, the citations in the identified literature reviews were analyzed to find any original studies that were not captured in the initial search.

Data were extracted from the included articles by one reviewer (ZL) and subsequently verified by a second reviewer (CZ). Any discrepancies were discussed and resolved with a third reviewer (HZ). The data collected comprised authors, sample size, study design, year of publication, details of the intervention (exercise type), and outcomes (BDNF levels). In certain studies, graphs were used to extract data with the WebPlotDigitizer (Rohatgi, 2011) online tool, eliminating the need to reach out to the authors for more details. Following data extraction, a third reviewer (HZ) confirmed the data’s completeness and accuracy. Furthermore, prior to data extraction, comprehensive exercise was provided to ensure that team members were proficient in the software used and had a thorough understanding of the subject matter (Young et al., 2012).

### 2.6 Statistical analysis

Data analysis was performed using Review Manager 5.4 (Cochrane Collaboration, Oxford, UK) and Stata SE V.15 software (Stata Corp). Initially, all the data are converted to a consistent unit (ng/mL), followed by calculating the mean difference between pre-intervention values and post-intervention values. The *I* ^2^ statistic was used to evaluate heterogeneity, with an *I* ^2^ value of 0%–40% signifying “low heterogeneity,” 30%–60% indicating “moderate heterogeneity,” 50%–90% showing “substantial heterogeneity,” and 75%–100% denoting “high heterogeneity,”' while taking into account the related p-values and 95% confidence intervals (Higgins et al., 2003).

Statistical analyses included comparisons of continuous variables. Crucial information was extracted from each study, including mean BDNF concentrations, standard deviations, and the number of participants in the intervention group pre-intervention and post-intervention. Either a fixed-effects model or a random-effects model was chosen according to the heterogeneity level. The Mantel-Haenszel method was used for a fixed-effects model when the heterogeneity test was not significant (Page et al., 2021). Conversely, a random-effects model was used when there was significant statistical heterogeneity (*I* ^2^ ≥ 50% or *p* < 0.1) (Mao et al., 2015). Moreover, when there was significant heterogeneity (*I* ^2^ > 50%), subgroup and sensitivity analyses were performed to clarify the results (Moher et al., 2015). To evaluate the magnitude of the effect, standardized mean differences (SMD) and 95% confidence intervals (CI) were determined. As per Cohen et al., effect sizes are categorized as small, medium, and large when they are 0.2, 0.5, and 0.8, respectively (Cohen, 1988). During sensitivity analysis, the summary estimate’s robustness was determined by sequentially eliminating each study from the included literature (Shamseer et al., 2015).

## 3 Results

### 3.1 Study selection

**FIGURE 1:**
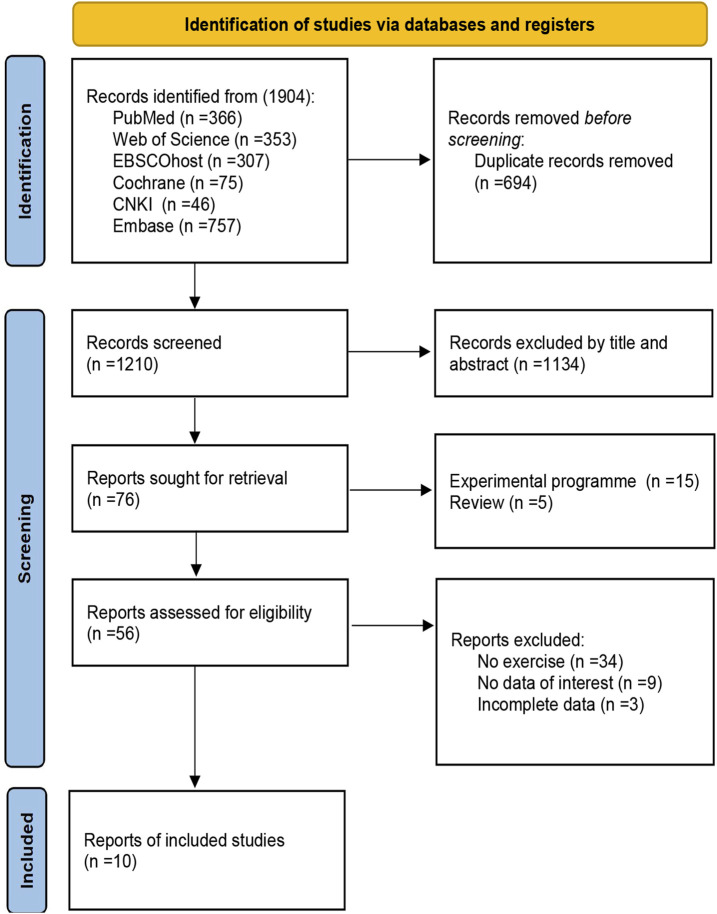
Study selection flowchart in PRISMA.

Figure 1 illustrates the study selection process. A total of 1,904 articles were obtained from the database search, and 694 duplicate articles were excluded. A total of 1,134 articles were excluded after examining their titles and abstracts. Among the remaining 76 articles, 15 were experimental protocols, 5 were reviews, 34 were non-exercise interventions, 9 had irrelevant experimental data, and 3 were incomplete data. 66 studies were excluded in total due to not meeting the inclusion criteria. Ultimately, 10 articles were included in the review (Brinkmann et al., 2017; Cokar et al., 2022; Luo et al., 2022; Babaei et al., 2023; Ghodrati et al., 2023; Goulet et al., 2023; Ploydang et al., 2023; Silveira-Rodrigues et al., 2023a; Silveira-Rodrigues et al., 2023b; Donyaei et al., 2024).

### 3.2 Characteristics of the included studies

Among the ten articles included in the analysis, Brinkmann et al.’s study included two independent intervention measures: cycling and aerobic exercise (Brinkmann et al., 2017). Similarly, Silveira-Rodrigues et al.’s study included two independent intervention measures: aerobic exercise and resistance training (Silveira-Rodrigues et al., 2023a). In Goulet’s study, two aerobic exercise intervention trials were conducted at temperatures of 16 °C and 32 °C (Goulet et al., 2023). Therefore, among the 10 included papers, there were a total of 13 studies. One study was conducted in China (Luo et al., 2022), three in Iran (Babaei et al., 2023; Ghodrati et al., 2023; Donyaei et al., 2024), one in Germany (Brinkmann et al., 2017), one in Turkey (Cokar et al., 2022), one in Canada (Goulet et al., 2023), one in Thailand (Ploydang et al., 2023), and two in Brazil (Silveira-Rodrigues et al., 2023a; Silveira-Rodrigues et al., 2023b). With sample sizes varying from 4 to 64, these studies collectively involved 206 people diagnosed with T2DM. A total of 6 studies reported the duration of diabetes in the samples. Among these, 4 studies had sample durations of no more than 10 years (Brinkmann et al., 2017; Luo et al., 2022; Ghodrati et al., 2023), while two studies had sample durations exceeding 10 years (Cokar et al., 2022; Silveira-Rodrigues et al., 2023b). Regarding the type of BDNF samples, 10 studies analyzed serum BDNF (Brinkmann et al., 2017; Cokar et al., 2022; Luo et al., 2022; Babaei et al., 2023; Ghodrati et al., 2023; Goulet et al., 2023; Ploydang et al., 2023; Donyaei et al., 2024), while 3 studies analyzed plasma BDNF (Silveira-Rodrigues et al., 2023a; Silveira-Rodrigues et al., 2023b). Regarding the timing of BDNF sampling, seven studies collected blood samples immediately after exercise (Brinkmann et al., 2017; Cokar et al., 2022; Goulet et al., 2023; Silveira-Rodrigues et al., 2023a), while six studies collected blood samples at a non-immediate time point after exercise (Luo et al., 2022; Babaei et al., 2023; Ghodrati et al., 2023; Ploydang et al., 2023; Silveira-Rodrigues et al., 2023b; Donyaei et al., 2024). Of the studies reviewed, eight investigated aerobic exercise interventions (Brinkmann et al., 2017; Cokar et al., 2022; Luo et al., 2022; Goulet et al., 2023; Ploydang et al., 2023; Silveira-Rodrigues et al., 2023a), one focused on resistance exercise (Silveira-Rodrigues et al., 2023a),and four adopted combined exercise methods, which involved aerobic exercise paired with resistance exercise, aerobic exercise paired with resistance exercise, and balance exercises (Babaei et al., 2023; Ghodrati et al., 2023; Silveira-Rodrigues et al., 2023b; Donyaei et al., 2024). Six studies assessed acute exercise interventions (Brinkmann et al., 2017; Goulet et al., 2023; Silveira-Rodrigues et al., 2023a), while seven explored the effects of chronic exercise (Cokar et al., 2022; Luo et al., 2022; Babaei et al., 2023; Ghodrati et al., 2023; Ploydang et al., 2023; Silveira-Rodrigues et al., 2023b; Donyaei et al., 2024). Notably, all seven chronic exercise studies incorporated both aerobic and combined exercise modalities. The length of the interventions spanned from around 8–24 weeks, with exercise conducted 3 to 5 times per week, and each session lasting from 35 to 65 min. BDNF levels were evaluated using ELISA techniques, with the necessary kits being sourced from China and the United States. The studies consisted of seven randomized controlled trials (Brinkmann et al., 2017; Babaei et al., 2023; Ghodrati et al., 2023; Ploydang et al., 2023; Silveira-Rodrigues et al., 2023b; Donyaei et al., 2024), while six were non-randomized controlled trials (Cokar et al., 2022; Luo et al., 2022; Goulet et al., 2023; Silveira-Rodrigues et al., 2023a) (Table 1).

**TABLE 1:** Characteristics of studies included in the meta-analysis.

Study	Country	Sample Size(n)	female (%)	Age (yr)	Time of T2DM diagnosis (yr)	BDNF sample	BDNF baseline level (SD) (ng/mL)	BDNF sampling time	BDNF measurement	Exercise Types	Exercise content	Intensity	Duration	Volume	Research findings
Donyaei et al. (2024)	Iran	17	100	61.3 ± 5.7	NA	Serum	0.22 ± 0.01	48 h after the last intervention	ELISA	Combined exercise	Circuit resistance exercise and treadmill exercise	Resistance exercise: 50%–70% 1RM Aerobic exercise: 50%–70% HRR	12 Week	60 min × 3 sessions/week	Rise
Brinkmann et al. (2017)	Germany	4	0	71 ± 4	6 ± 3	Serum	34.38 ± 10.12	Collect blood immediately after the intervention ends	ELISA	Aerobic exercise	Bicycle	rpm = ∼65	Acute	30 min	Rise
Brinkmann et al. (2017)	Germany	4	0	71 ± 4	6 ± 3	Serum	31.19 ± 9.98	Collect blood immediately after the intervention ends	ELISA	Aerobic exercise	“Wii Fit Plus” system plays virtual sports games	No report	Acute	30 min	no significant change
Cokar et al. (2022)	Turkey	16	100	57.5 ± 7.4	11.3 ± 5.04	Serum	0.6 ± 0.4	Collect blood immediately after the intervention ends	ELISA	Aerobic exercise	Treadmill Walking	50%–70% HRR	12 Week	40 min × 3 sessions/week	no significant change
Silveira-Rodrigues et al. (2023a)	Brazil	9	NA	63.8 ± 7.12	NA	Plasma	0.45 ± 0.15	Collect blood immediately after the intervention ends	ELISA	Aerobic exercise	Treadmill Walking	90%–95% of maximum walking speed	Acute	40 min	Rise
Silveira-Rodrigues et al. (2023a)	Brazil	9	NA	63.8 ± 7.12	NA	Plasma	0.51 ± 0.15	Collect blood immediately after the intervention ends	ELISA	Resistance exercise	Upper and lower limb resistance exercise	70% 10RM	Acute	40 min	no significant change
Silveira-Rodrigues et al. (2023b)	Brazil	16	62.50	63.9 ± 7.7	11.3 ± 7.7	Plasma	0.18 ± 0.09	72 h after the last intervention	ELISA	Combined exercise	Aerobic exercise: Treadmill Running Resistance exercise: Six resistance exercises for the upper and lower limbs	No report	8 Week	60 min × 3 sessions/week	no significant change
Luo et al. (2022)	China	64	42.19	66.32 ± 3.43	4.07 ± 1.91	Serum	4.97 ± 3.05	The morning after the last training session	ELISA	Aerobic exercise	Walking on flat ground	55–70 steps per minute	24 Week	35–45 min × 5 sessions/week	Rise
Ghodrati et al. (2023)	Iran	12	100	58.8 ± 1.5	6.3 ± 1.9	Serum	3.4 ± 1.7	48 h after the last intervention	ELISA	Combined exercise	Running, resistance exercise, and balance exercises	Aerobic exercise: 55%–75% HRR Resistance exercise: 65%–85% 1RM Balance exercise: 20–40 s	12 Week	65 min × 3 sessions/week	no significant change
Goulet et al. (2023)	Canada	9	0	60 ± 5	NA	Serum	1.32 ± 0.54	Collect blood immediately after the intervention ends	ELISA	Aerobic exercise	Walking on a treadmill at 16 °C	42.4%HRR	Acute	180 min	no significant change
Goulet et al. (2023)	Canada	9	0	60 ± 5	NA	Serum	1.47 ± 0.92	Collect blood immediately after the intervention ends	ELISA	Aerobic exercise	Walking on a treadmill at 32 °C	81.4%HRR	Acute	180 min	Rise
Babaei et al. (2023)	Iran	21	100	72.66 ± 6.31	NA	Serum	1.43 ± 0.01	36 h after the last intervention	ELISA	Combined exercise	Treadmill exercise, Multi-Stage Resistance Output Measurement (MSROM), and other exercise activities	Aerobic exercise: 55%–75% HRR Resistance exercise: No report	8 Week	60 min × 3 sessions/week	Rise
Ploydang et al. (2023)	Thailand	16	68.75	68.9 ± 3.7	NA	Serum	0.23 ± 0.03	After fasting for 12 h	ELISA	Aerobic exercise	Nordic Water Walking	40%-60% HRR	12 Week	40 min × 3 sessions/week	Rise

### 3.3 Risk of bias

**FIGURE 2:**
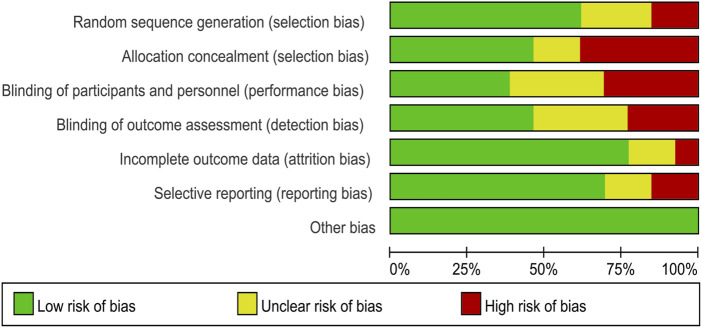
Risk of bias graph.

The risk of bias in the studies included in this analysis was assessed using the Cochrane Risk of Bias tool. For the domain of random sequence generation, eight studies (Brinkmann et al., 2017; Goulet et al., 2023; Ploydang et al., 2023; Silveira-Rodrigues et al., 2023a; Donyaei et al., 2024) were deemed to have a low risk of bias, three studies (Babaei et al., 2023; Ghodrati et al., 2023; Silveira-Rodrigues et al., 2023b) were assessed as having an unclear risk, and two studies (Cokar et al., 2022; Luo et al., 2022) were identified as having a high risk. Regarding allocation concealment, six studies (Brinkmann et al., 2017; Ghodrati et al., 2023; Silveira-Rodrigues et al., 2023a; Donyaei et al., 2024) were categorized as low risk, two studies (Cokar et al., 2022; Babaei et al., 2023) as unclear risk, and five studies (Luo et al., 2022; Goulet et al., 2023; Ploydang et al., 2023; Silveira-Rodrigues et al., 2023b) as high risk. In terms of performance bias, five studies (Brinkmann et al., 2017; Cokar et al., 2022; Silveira-Rodrigues et al., 2023a) were determined to have a low risk, four studies (Luo et al., 2022; Ghodrati et al., 2023; Goulet et al., 2023) were considered to have an unclear risk, and four studies (Babaei et al., 2023; Ploydang et al., 2023; Silveira-Rodrigues et al., 2023b; Donyaei et al., 2024) were classified as high risk. For detection bias, six studies (Cokar et al., 2022; Luo et al., 2022; Babaei et al., 2023; Ploydang et al., 2023; Silveira-Rodrigues et al., 2023b; Donyaei et al., 2024) were classified as low risk, four studies (Brinkmann et al., 2017; Silveira-Rodrigues et al., 2023a) as unclear risk, and three studies (Ghodrati et al., 2023; Goulet et al., 2023) as high risk. Concerning attrition bias, ten studies (Brinkmann et al., 2017; Cokar et al., 2022; Luo et al., 2022; Babaei et al., 2023; Ghodrati et al., 2023; Goulet et al., 2023; Ploydang et al., 2023; Donyaei et al., 2024) were rated as low risk, two studies (Silveira-Rodrigues et al., 2023a) as unclear risk, and one study (Silveira-Rodrigues et al., 2023b) as high risk. In the domain of reporting bias, nine studies (Cokar et al., 2022; Luo et al., 2022; Babaei et al., 2023; Ghodrati et al., 2023; Ploydang et al., 2023; Silveira-Rodrigues et al., 2023a; Silveira-Rodrigues et al., 2023b; Donyaei et al., 2024) were classified as low risk, two studies (Brinkmann et al., 2017) as unclear risk, and two studies (Goulet et al., 2023) as high risk. Finally, all 13 studies (Brinkmann et al., 2017; Cokar et al., 2022; Luo et al., 2022; Babaei et al., 2023; Ghodrati et al., 2023; Goulet et al., 2023; Ploydang et al., 2023; Silveira-Rodrigues et al., 2023a; Silveira-Rodrigues et al., 2023b; Donyaei et al., 2024) were assessed as having a low risk concerning other biases (Figure 2).

### 3.4 Publication bias

**FIGURE 3:**
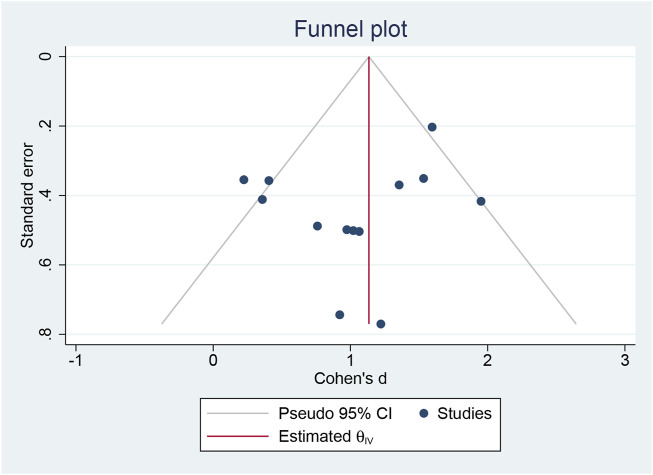
Publication bias.

A funnel plot and Egger’s test (Egger et al., 1997) were employed to assess publication bias among the 13 experimental studies. The results indicated no significant publication bias regarding BDNF concentration (*p* = 0.085) (Figure 3).

### 3.5 Meta-analysis

In this systematic review and meta-analysis, we aimed to investigate the effects of exercise on BDNF levels in middle-aged and elderly patients with T2DM. Despite the small sample sizes in some studies and high or unclear risks of bias in allocation concealment and blinding, we included these studies in the analysis. First, given the scarcity of research on the effects of exercise on BDNF levels in middle-aged and elderly T2DM patients, these studies provide valuable data that enhance the statistical power of this analysis. Second, these studies reflect common research designs and implementation practices in this field, and their inclusion helps assess the prevalence and potential limitations of these designs in real-world applications. Additionally, for studies with high or unclear risks, this study conducted sensitivity analyses to exclude high-risk studies and used a random-effects model to account for heterogeneity among studies, thereby enhancing the reliability of the results.

#### 3.5.1 The effects of exercise on BDNF

**FIGURE 4:**
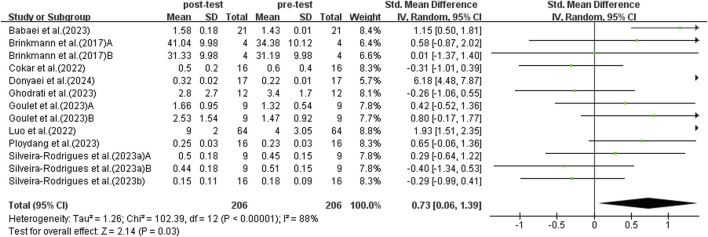
Forest plot of effects of exercise on BDNF.

Figure 4 shows a strong link between exercise and higher BDNF levels in middle-aged and older adults with T2DM. Due to significant study variability (*I* ^2^ = 88%, *p* < 0.00001), a random-effects model was used. Exercise positively impacts BDNF levels in middle-aged and older adults with T2DM (SMD = 0.73, 95% CI: 0.06 to 1.39, *p* = 0.03). Sensitivity analyses excluding individual studies or those with smaller samples showed no significant changes.

#### 3.5.2 Subgroup analysis: aerobic exercise and combined exercise

This study conducted subgroup analyses comparing single exercise modalities and combined exercise interventions. The majority of the studies incorporated aerobic and combined exercise interventions, with only a single study using resistance exercise. Consequently, the resistance exercise study was excluded from the current subgroup analysis. The subgroup analysis was based on 12 studies focusing on aerobic and combined exercise interventions. A random-effects model was used for analysis because of substantial heterogeneity between studies (*I* ^2^ = 87%, *p* < 0.00001).

Sensitivity analysis revealed high heterogeneity (*I* ^2^ = 73%, *p* = 0.0005) in aerobic exercise studies. Upon excluding Luo’s trial, inter-study heterogeneity was eliminated (*p* = 0.52, *I* ^2^ = 0%). After this exclusion, the SMD = 0.31, with a 95% CI: −0.04 to 0.65, and the result was not statistically significant (*p* = 0.08). Further investigation identified significant methodological variations. Luo et al.'s study used ground walking as the primary exercise intervention, while other included aerobic exercise studies were based on treadmill training. Although treadmill training and ground walking are both forms of walking exercise, they exhibit significant differences in exercise environment, psychological factors, and biomechanical characteristics (Lee and Hidler, 2008). Existing research indicates that the two walking modes differ significantly in muscle activation patterns, joint torque, and joint power (Lee and Hidler, 2008). Additionally, a meta-analysis noted that relative oxygen consumption and step frequency are significantly higher during treadmill exercise compared to ground walking (Vickery-Howe et al., 2023). These differences may lead to biases in the assessment of exercise intervention effects. In addition to exercise mode, there are also differences in intensity measurement methods across studies. Luo et al. used a maximum oxygen uptake (THR)-based intensity standard, while other studies employed heart rate reserve (HRR) as an assessment metric. Although both methods can be used for exercise intensity assessment, they have distinct differences in their calculation formulas and practical applications (Chen et al., 2022). Specifically, the THR method is simple to calculate but does not account for individual differences in resting heart rate, potentially leading to inaccuracies; in contrast, the HRR method incorporates resting heart rate data, enabling more precise reflection of an individual’s actual exercise intensity (Kirkham et al., 2013). Therefore, Luo’s study was excluded from the subgroup analysis.

**FIGURE 5:**
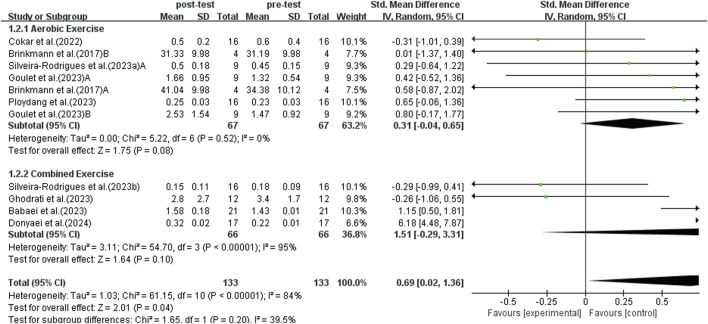
Forest plot of aerobic exercise and combined exercise on BDNF.

The subgroup analysis comprised 11 studies after excluding Luo’s study. As illustrated in Figure 5, aerobic exercise did not significantly elevate BDNF levels in middle-aged and older adults with T2DM (SMD = 0.31, 95% CI: −0.04 to 0.65, *p* = 0.08). Combined exercise also did not significantly increase BDNF levels in middle-aged and elderly patients with T2DM (SMD = 1.51, 95% CI: −0.29 to 3.31, *p* = 0.10).

#### 3.5.3 Subgroup analysis: acute exercise and chronic exercise

**FIGURE 6:**
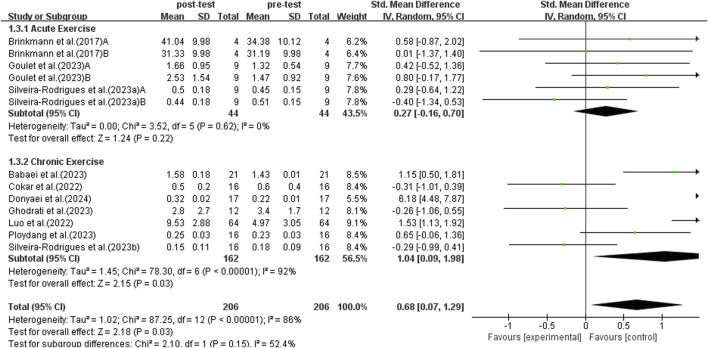
Forest plot of acute exercise and chronic exercise on BDNF.

Results from a subgroup analysis of 13 studies examining acute and chronic exercise are depicted in Figure 6. A random-effects model was used for analysis because the studies demonstrated high heterogeneity (*I* ^2^ = 86%, *p* < 0.00001). Sensitivity analysis indicated no significant differences upon the sequential exclusion of individual studies or those with small sample sizes. The findings from the subgroup analysis revealed that acute exercise did not significantly enhance BDNF levels in middle-aged and older adults with T2DM (SMD = 0.27, 95% CI: −0.16 to 0.70, *p* = 0.22). In contrast, sustained exercise was linked to a notable rise in BDNF levels among middle-aged and older individuals with T2DM (SMD = 1.04, 95% CI: 0.09 to 1.98, *p* = 0.03). Thus, chronic exercise exhibited a more pronounced effect on the enhancement of BDNF levels.

#### 3.5.4 Subgroup analysis: weekly exercise duration

**FIGURE 7:**
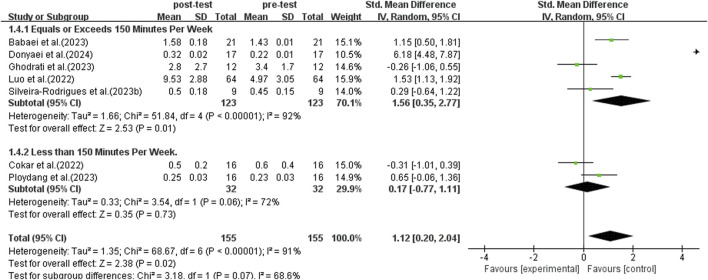
Forest plot of weekly exercise duration on BDNF.

Subgroup analyses were conducted following World Health Organization recommendations, comparing individuals exercising for 150 min or more per week to those exercising less than 150 min (Bull et al., 2020). This analysis incorporated data from seven studies, as illustrated in Figure 7. A random-effects model was used for the analysis because of the significant heterogeneity among the studies (*I* ^2^ = 91%, *p* < 0.00001). Conducting a sensitivity analysis by sequentially excluding individual studies or those with small sample sizes did not reveal any significant differences. The subgroup analysis indicated that engaging in 150 min or more of exercise per week significantly elevated BDNF levels in middle-aged and older adults with T2DM (SMD = 1.56, 95% CI: 0.35 to 2.77, *p* = 0.01). On the other hand, exercising for less than 150 min per week did not significantly enhance BDNF levels in middle-aged and older adults with T2DM (SMD = 0.17, 95% CI: −0.77 to 1,011, *p* = 0.73). As a result, participating in over 150 min of exercise per week has a significantly greater impact on increasing BDNF levels.

#### 3.5.5 Subgroup analysis: time of blood collection

**FIGURE 8:**
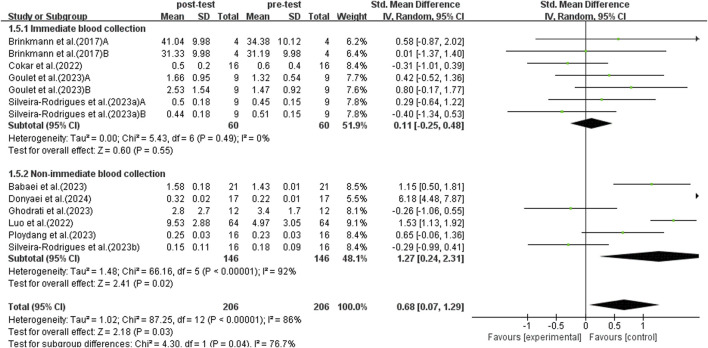
Forest plot of weekly exercise duration on BDNF.

According to research, there are differences in BDNF levels detected in blood samples collected at different times after exercise (Brunelli et al., 2012). The study conducted a subgroup analysis of blood collection times post-exercise to investigate the effects of immediate *versus* non-immediate blood collection. This analysis included data from 13 studies, as shown in Figure 8. Due to significant heterogeneity among studies (*I* ^2^ = 86%, *p* < 0.00001), a random-effects model was used for analysis. Sensitivity analyses were conducted by sequentially excluding individual studies or studies with smaller sample sizes, and no significant differences were observed. The subgroup analysis results indicated that immediate blood collection after exercise did not show a significant increase in BDNF levels in elderly patients with T2DM (SMD = 0.11, 95% CI: −0.25 to 0.48, *p* = 0.55). On the other hand, non-immediate blood sampling after exercise detected a significant increase in BDNF levels in elderly patients with T2DM (SMD = 1.27, 95% CI: 0.24 to 2.31, *p* = 0.02). Therefore, the timing of blood sampling affects the test results, and delayed sampling more accurately reflects the promotional effect of exercise on BDNF in elderly populations with T2DM.

#### 3.5.6 Subgroup analysis: BDNF sample

**FIGURE 9:**
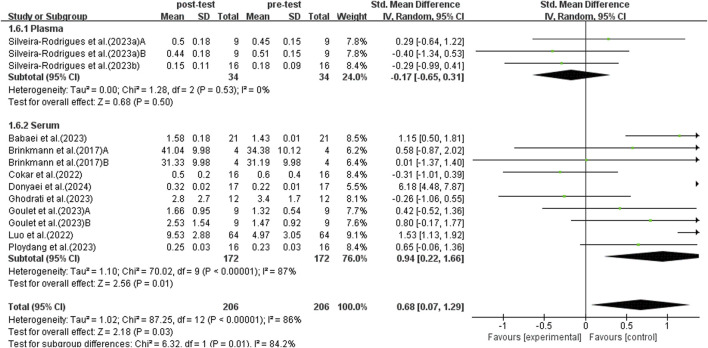
Forest plot of weekly exercise duration on BDNF.

BDNF can be detected in plasma and serum. Previous studies have shown that there are differences in BDNF concentrations detected in plasma and serum (Radka et al., 1996). Therefore, a subgroup analysis was conducted on plasma and serum, incorporating data from 13 studies, as shown in Figure 9. Due to significant heterogeneity among studies (*I* ^2^ = 86%, *p* < 0.00001), a random-effects model was used for analysis. Sensitivity analyses were conducted by sequentially excluding individual studies or studies with smaller sample sizes, and no significant differences were found. Subgroup analysis indicated that trials using plasma did not detect a significant increase in BDNF levels in elderly patients with T2DM (SMD = −0.17, 95% CI: −0.65 to 0.31, *p* = 0.50). On the other hand, trials using serum for detection found a significant increase in BDNF levels in elderly patients with T2DM (SMD = 0.94, 95% CI: 0.22 to 1.66, *p* = 0.01). Therefore, the choice of plasma or serum for detection influences the results, with serum BDNF detection having a more significant impact on the trend of BDNF elevation following exercise intervention.

#### 3.5.7 Subgroup analysis: different stages of diabetes

The duration of diabetes is positively associated with the risk of various diabetes-related complications. Studies have shown that patients with T2DM who have had the disease for ≥10 years have higher rates of complications and mortality than those with a disease duration of <10 years (Huang et al., 2014). Therefore, the study conducted a subgroup analysis based on disease duration of ≥10 years and <10 years, including a total of six studies. Due to significant heterogeneity among studies (*I* ^2^ = 87%, *p* < 0.00001), a random-effects model was used for analysis.

After sensitivity analysis, studies with a duration of less than 10 years showed higher heterogeneity (*I* ^2^ = 84%, P < 0.00001). After excluding Luo et al.'s study, there was no heterogeneity among the studies (*p* = 0.61, *I* ^2^ = 0%). After this exclusion, the SMD = −0.04, with a 95% CI: −0.67 to 0.58, and the result was not statistically significant (*p* = 0.89). Further analysis revealed that the BMI of the samples in Luo et al.'s study was lower than that in other studies. A lower BMI may amplify the BDNF response. In addition, the intensity of the exercise regimen in Luo et al.'s study was lower than that in other studies. The differences in BMI and exercise intensity further increased the variability of the results. Therefore, Luo’s study was excluded from the subgroup analysis.

**FIGURE 10:**
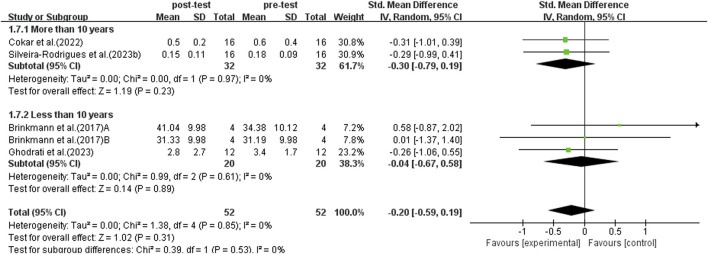
Forest plot of weekly exercise duration on BDNF.

After excluding Luo et al.'s study, the subgroup analysis included five studies. As shown in Figure 10, no significant heterogeneity persisted among studies (*I* ^2^ = 0%, *p* = 0.85), and no significant increase in BDNF levels was observed in patients with a disease duration of ≥10 years (SMD = −0.30, 95% CI: −0.79 to 0.03, *p* = 0.19). Patients with a disease duration of <10 years showed a significant increase in BDNF levels (SMD = −0.04, 95% CI: −0.67 to 0.58, *p* < 0.00001). Therefore, exercise has a more significant BDNF-enhancing effect in middle-aged and elderly patients with T2DM who have a shorter disease duration (<10 years).

### 3.6 Clinical effect

To investigate the effect of exercise on improving BDNF levels in middle-aged and older adults with T2DM, we examined the minimum clinically important difference (MCID) in the included studies. The MCID represents the minimum threshold for clinically meaningful improvement from the patient’s perspective (Bloom et al., 2023). Therefore, this indicator holds significant clinical importance for validating treatment efficacy. Currently, the primary methods for determining the MCID are anchor-based and distribution-based approaches (Copay et al., 2007). This study will use the distribution-based method to calculate the MCID based on effect size. According to Cohen et al., an effect size of 0.2 is considered a small effect, 0.5 is a moderate effect, and 0.8 or higher is a large effect (Cohen, 1988). Some studies suggest that a change score with an effect size of 0.2 is equivalent to the MCID, calculated using the formula MCID (ES-based) = 0.2 × SD (BaselineScore) (Klukowska et al., 2024). If patients show only slight improvement in post-intervention outcome measures, the calculated result will be smaller than the pooled effect. The total pooled effect size for exercise on MCID of BDNF in older adults with T2DM is SMD = 0.73, 95% CI: 0.06 to 1.39. As shown in Table 2, only two studies exceeded the MCID after exercise intervention.

**TABLE 2:** MCID for each study.

Study	MCID	Total combined effect size
Brinkmann et al. (2017)	2.024	0.73 [0.06, 1.39]
Brinkmann et al. (2017)	1.996	

## 4 Discussion

This systematic review and meta-analysis encompasses 13 studies involving a total of 206 middle-aged patients diagnosed with T2DM, aiming to investigate the impact of exercise on BDNF levels within this population. The study results indicate that exercise significantly increases BDNF levels in elderly patients diagnosed with T2DM. Subgroup analysis shows that chronic exercise has a more significant effect on BDNF levels in patients with type 2 diabetes than acute exercise. Notably, engaging in 150 min or more of exercise per week was associated with a significant increase in BDNF levels in elderly patients with type 2 diabetes. Additionally, this systematic review and meta-analysis assessed the effects of different exercise parameters on BDNF levels in middle-aged and elderly patients with type 2 diabetes, while also considering the influence of blood collection timing, different blood samples, and diabetes duration. Subgroup analysis results indicated that delayed blood collection better reflected the increase in BDNF levels compared to immediate blood collection after exercise. Measuring BDNF levels in serum showed a more significant effect compared to measuring BDNF levels in plasma.

### 4.1 Impact of exercise on BDNF levels in middle-aged and older adults with T2DM

Within the field of diabetes research, BDNF is considered a crucial element in enhancing glucose metabolism and insulin sensitivity, serving as an essential mediator in the pathophysiological mechanisms underlying T2DM (Tsuchida et al., 2001; Yamanaka et al., 2007). To investigate the effect of exercise on improving BDNF levels in older adults with T2DM, we calculated the MCID in each independent study. However, the majority of studies did not exceed the MCID. Due to the limitations of the studies, we were restricted to using a distribution-based method to calculate the MCID. However, this method ignores patients’ subjective assessments of “meaningful change” and may underestimate the true magnitude of benefits that are critical to patients. In contrast, patient-centered anchoring methods can directly reflect individual perceptions. Although the distribution-based method can calculate the MCID, it typically yields smaller estimates (Copay et al., 2008). Therefore, some studies suggest validating the MCID using other calculation methods, preferably those based on anchoring (Klukowska et al., 2024). It is worth noting that only two studies reached the pre-specified MCID threshold. Although the pooled effect showed that exercise intervention statistically significantly increased BDNF levels in middle-aged and older adults with T2DM, most training programs still failed to achieve the minimum clinical benefit that patients could perceive. The discrepancy between statistical significance and clinical relevance highlights the need for further research. Future studies could explore optimizing exercise intensity, duration, or intervention combinations to further enhance the minimum clinically important improvement that is clinically perceptible for middle-aged and older adults with T2DM. In addition, although we used funnel plots and Egger’s test, there was no significant publication bias (*p* = 0.085). However, due to the small number of studies included, this means that even if the Egger’s test p-value is >0.05, its statistical power may still be insufficient (Furuya-Kanamori et al., 2020). Our study focuses on the impact of exercise on BDNF levels in older adults with T2DM. BDNF has been identified as a novel protein induced by muscle contraction and synthesized in skeletal muscle cells, facilitating lipid oxidation *via* an AMPK-dependent pathway (Pedersen, 2011). Nevertheless, the reduction in muscle mass observed in elderly T2DM patients leads to inadequate BDNF production, which may exacerbate diabetic complications. Engaging in exercise has been proven to greatly enhance muscle strength in individuals with diabetes and boost BDNF expression (Ghodrati et al., 2023). Our research further corroborates this perspective, demonstrating that exercise effectively elevates BDNF levels in middle-aged and older adults with T2DM populations. In addition, long-term exercise can improve insulin sensitivity and vascular endothelial function, reducing the inhibition of BDNF expression by hyperglycemia (Cai et al., 2022). Therefore, regular exercise can effectively increase BDNF concentration in muscle tissue, thereby slowing down the pathological process and improving metabolic function.

Changes in BDNF levels are significantly associated with various clinical outcomes. As the disease progresses, patients with T2DM may experience significant neurophysiological and structural changes in their peripheral systems and central nervous systems (Sloan et al., 2021), thereby increasing the risk of cognitive dysfunction, such as Alzheimer’s disease and diabetic encephalopathy (Luo et al., 2024). The neuroprotective effects of BDNF have been validated in multiple studies. Changes in the intracellular transport and secretion of BDNF play a crucial role in brain regions associated with cognitive function, as demonstrated by relevant research. Specifically, higher levels of BDNF are associated with larger hippocampal volume (Erickson et al., 2012), can inhibit diabetes-related cortical cell apoptosis (Cheng and Lee, 2022), and enhance neural plasticity in the frontal cortex (Marqués-Iturria et al., 2014). Additionally, clinical observations have revealed a significant negative correlation between hyperglycemia and insulin resistance severity and circulating BDNF levels (Krabbe et al., 2007). Animal experiments further support BDNF’s metabolic regulatory role. In a diabetic rat model, exogenous BDNF intervention significantly reduced serum insulin and glucose levels and improved metabolic regulation (Tonra et al., 1999). This includes improving insulin resistance in rat livers, enhancing pancreatic β-cell function, and increasing peripheral tissue glucose utilization, thereby optimizing blood glucose control (Kuroda et al., 2003; Yamanaka et al., 2007; Jiménez-Maldonado et al., 2017). Among these, skeletal muscle activity-induced BDNF secretion can enhance insulin secretion and reduce blood glucose levels during hyperglycemia (Fulgenzi et al., 2020)]. A review report indicates that low levels of BDNF may be a risk factor for diabetic neurovascular complications, and exogenous BDNF supplementation may become a potential therapeutic strategy (Rozanska et al., 2020). Therefore, BDNF plays a crucial role in maintaining cognitive function and improving diabetic metabolism.

### 4.2 Effects of aerobic exercise and combined exercise on BDNF levels in middle-aged and older adults with T2DM

Aerobic exercise is widely recognized for its health benefits, including the effective reduction of risks associated with cardiovascular diseases, diabetes, and central nervous system disorders (Ribeiro et al., 2021). Erickson et al. conducted a study assessing the impact of aerobic exercise on hippocampal volume and serum BDNF levels in 60 elderly participants. Their findings demonstrated that a 6-month intervention led to an increase in both hippocampal volume and BDNF levels (Erickson et al., 2011). Conversely, findings from different studies imply that aerobic exercise does not substantially boost BDNF levels in elderly people with T2DM (Cokar et al., 2022). The results of this study are consistent with prior research, indicating that various factors may influence the impact of aerobic exercise on BDNF levels in middle-aged and older adults with T2DM. A substantial body of literature has demonstrated that exercise can increase BDNF levels in children and adolescents (Jeon and Ha, 2017; Mora-Gonzalez et al., 2019; Wunram et al., 2021), implying that age may affect the effectiveness of aerobic exercise in boosting BDNF levels. Moreover, research has identified an association between BDNF levels and muscle strength (Guilherme et al., 2022). Although aerobic exercise markedly improves cardiorespiratory fitness and metabolic status in middle-aged and older adults with T2DM, its impact on muscle strength and mass is limited. Consequently, more studies are needed to explore how aerobic exercise affects BDNF levels in this group.

Engaging in combined exercise markedly improves physiological functions, thereby enhancing cardiovascular, nervous, and muscular capabilities (Bossers et al., 2015; Schroeder et al., 2019). This study focused on an integration of aerobic and resistance exercise modalities. Evidence suggests that combined exercise is more efficacious than single-mode exercise in increasing BDNF levels (Donyaei et al., 2024). Different exercise modalities regulate BDNF secretion by activating distinct signaling pathways. Research indicates that aerobic exercise primarily promotes BDNF release by activating the AMP-activated protein kinase (AMPK) signaling pathway (Zhou et al., 2025), while resistance exercise relies on the mammalian target of rapamycin (mTOR) signaling pathway to exert its effects (Atherton et al., 2005). However, these two pathways exhibit antagonistic effects. During aerobic exercise, AMPK activation can inhibit mTOR signaling (Watson and Baar, 2014). Therefore, within the same training session, AMPK activation following aerobic exercise may suppress mTOR signaling during subsequent resistance training, which may explain why combined exercise failed to significantly increase BDNF levels in some studies. Additionally, a hyperglycemic environment may further interfere with AMPK signaling pathway activation, thereby reducing BDNF release (Wang et al., 2019). Therefore, future studies targeting middle-aged and older adults with T2DM should optimize the exercise sequence, rest intervals, and intensity ratios of combined training.

### 4.3 Effects of acute and chronic exercise on BDNF levels in middle-aged and older adults with T2DM

Studies indicate that both acute and chronic exercise can augment the production of BDNF in humans. (Etnier et al., 2016; Enette et al., 2020). However, these two forms of exercise exert distinct effects on the elevation and maintenance of BDNF levels in middle-aged and older adults. Specifically, a study conducted by Laske et al. revealed that a single exercise session significantly increased BDNF levels in elderly participants (Laske et al., 2010). In contrast, research by Schmidt-Kassow observed that although BDNF levels rose significantly within 20 min post-exercise, they reverted to baseline within 10 min after the activity ceased (Schmidt-Kassow et al., 2012). This indicates that the elevation in BDNF levels resulting from acute exercise is temporary. Conversely, Erickson et al. reported a significant elevation in BDNF levels following 1 year of regular exercise in older adults (Erickson et al., 2011). However, research suggests that BDNF levels typically normalize within 3–4 weeks after stopping chronic exercise (Berchtold et al., 2010). In addition, BDNF is partly produced in the hippocampus of the human body. Long-term chronic exercise can limit the decline of the hippocampus caused by the normal aging process, thereby further improving the secretion of BDNF in the hippocampus (Erickson et al., 2009; Ten Brinke et al., 2015). As a result, chronic exercise seems to be more effective in maintaining higher BDNF levels in middle-aged and older adults with T2DM.

The effect of acute exercise on increasing BDNF levels in middle-aged and older adults with T2DM is unclear, though some studies indicate it may significantly enhance these levels (Brinkmann et al., 2017; Silveira-Rodrigues et al., 2023a), while others suggest no significant increase occurs (Goulet et al., 2023). Ross et al. propose that the degree of BDNF elevation after acute exercise may be linked to workout intensity, with higher intensity correlating with greater BDNF concentrations (Ross et al., 2019). In contrast, chronic exercise promotes adaptive physiological changes through sustained exercise. Specific thresholds of exercise volume are required to stimulate BDNF production in certain muscle groups (Jiménez-Maldonado et al., 2014); if these thresholds are not met, significant increases in BDNF levels may not occur. Erickson also suggests that the hippocampus, rich in BDNF, can increase in volume with prolonged exercise, leading to greater BDNF release and higher overall levels (Erickson et al., 2011). While acute exercise can temporarily boost BDNF expression through increased cerebral blood flow, these effects are not lasting (Steinberg et al., 2018). Chronic exercise is more effective at increasing BDNF levels in middle-aged and older adults because of its long-term cumulative effects.

### 4.4 Effects of weekly exercise duration on BDNF levels in middle-aged and older adults with T2DM

In middle-aged and older adults with T2DM, BDNF levels are typically lower than in younger adults. The disparity arises from aging and diseases like diabetes linked to endothelial dysfunction. Notably, an exercise regimen of less than 150 min per week may be insufficient to achieve significant improvements in BDNF levels. For example, Maass et al. found that older adults cycling three times a week for 30 min over 3 months saw no notable BDNF increase (Maass et al., 2016). In contrast, the study by Jeon et al. demonstrated a marked increase in BDNF levels among adolescents who undertook high-intensity aerobic exercise sessions of less than 30 min, four times a week for 12 weeks (Jeon and Ha, 2017). This response may be due to adolescents being in a growth phase, during which their nervous and metabolic systems are more active and responsive to exercise. Similarly, a study on rats showed that BDNF levels decrease with age, with older rats exhibiting lower levels than younger ones (Croll et al., 1998). Additionally, elderly individuals with T2DM often face metabolic disorders that impede BDNF synthesis and further reduce BDNF levels (Liu et al., 2022; Cefis et al., 2023; Moosaie et al., 2023). Therefore, increasing exercise, particularly on weekends, is essential for enhancing BDNF levels in middle-aged and older adults with T2DM.

Exercising for at least 150 min weekly can reduce the risk of T2DM and enhance metabolic health in middle-aged and older adults with the condition (Earnest et al., 2014; Boonpor et al., 2023; Liu et al., 2024). This level of exercise also creates a more favorable internal environment for BDNF synthesis. However, the precise amount and intensity of exercise needed to elevate BDNF levels in this population remain inconclusive, as studies vary in findings concerning exercise effects on BDNF levels. For example, Luo et al. observed significant increases in BDNF levels following a 24-week exercise intervention involving 64 middle-aged and older adults diagnosed with T2DM (Luo et al., 2022). In contrast, the study conducted by Silveira-Rodrigues et al. found no substantial elevation in BDNF levels among 16 middle-aged and older adults with T2DM after an 8-week exercise regimen performed thrice weekly (Silveira-Rodrigues et al., 2023b). Variations in these results might be affected by elements like the number of participants, the length of time with T2DM, and the details of the exercise regimen. Therefore, subsequent research should focus on analyzing the impact of varying exercise volumes and intensities on BDNF levels in middle-aged and older adults with differing durations of T2DM. Determining the optimal exercise dosage is crucial for enhancing overall health and maximizing therapeutic benefits for patients with T2DM.

### 4.5 Effects of time of blood collection on BDNF levels in middle-aged and older adults with T2DM

According to subgroup analysis results, there was a significant difference in the magnitude of BDNF changes between immediate and non-immediate blood sampling after exercise. This finding suggests that the exercise-induced BDNF peak may occur during the recovery period rather than immediately post-exercise. Existing studies on changes in BDNF levels after exercise have reported inconsistent results. In male patients with depression, BDNF levels were observed to decrease initially after aerobic exercise and then increase again after 60 min of rest (Gustafsson et al., 2009). Conversely, studies on resistance training in healthy populations showed that serum BDNF levels increased immediately after exercise but decreased below baseline levels after 60 min of recovery (Yarrow et al., 2010). This may be influenced by individual differences and variations in exercise intervention protocols. Metabolic state may also affect BDNF’s response to exercise. Compared to healthy individuals, central BDNF secretion in obese and T2DM patients may be inhibited by a chronic hyperglycemic environment (Krabbe et al., 2007). The aforementioned mechanisms may explain why, in the elderly T2DM population in this study, non-immediate blood collection resulted in a significant increase in BDNF levels. Additionally, in the subgroup analyses included, blood collection for acute exercise was performed post-exercise. The primary focus was on the acute effects of a single exercise session on BDNF in elderly T2DM patients. For chronic exercise, blood collection was mostly performed some time post-exercise, with the primary focus on the long-term effects of exercise on BDNF in elderly T2DM patients. It is worth noting that existing studies on the effects of acute exercise on BDNF levels in T2DM patients remain controversial. This may partially stem from differences in blood collection timepoints. In terms of long-term exercise interventions, some studies have shown that a 9-month program of aerobic, resistance, or combined training did not result in significant changes in BDNF levels in T2DM patients (Swift et al., 2012). Therefore, the complex release mechanism of BDNF is influenced by multiple interacting factors, including exercise intensity, duration, and blood collection timepoints.

### 4.6 Effects of BDNF sample on BDNF levels in middle-aged and older adults with T2DM

BDNF is widely distributed in the central nervous system and peripheral circulatory system. Clinical testing can detect BDNF in human plasma and serum samples, but its concentration varies significantly. Research indicates that platelets serve as the primary reservoir for BDNF and are closely associated with circulating BDNF levels (Serra-Millàs, 2016). Notably, BDNF levels in serum are 100 times higher than in plasma (Radka et al., 1996), which may be due to the release of BDNF by platelets during the clotting process (Fujimura et al., 2002). Plasma BDNF levels may be influenced by local muscle metabolism (Gilder et al., 2014). A study showed that muscle contraction significantly increases BDNF content and mRNA expression levels in muscle cells (Matthews et al., 2009). In a study of healthy humans, BDNF levels in both muscle and plasma can be increased through physical exercise, and differences in muscle fiber types can influence BDNF expression levels in muscle (Edman et al., 2024). Compared to serum BDNF levels, the recovery of plasma BDNF to near baseline values after exercise is slower (Gilder et al., 2014). Therefore, serum and plasma differ in composition and function, which may be one of the reasons for discrepancies in BDNF measurement results. Additionally, improper use of ELISA kits, as well as improper collection and processing of plasma and serum, may affect BDNF measurement results (Elfving et al., 2010). A study comparing six commercially available ELISA kits found that serum BDNF concentration detection is influenced by different ELISA kits (Polacchini et al., 2015). BDNF concentrations in plasma can be influenced by the method of blood sample processing, such as the shear force of the needle during blood collection, which can induce platelet degranulation, and even minor changes in room temperature and processing time may lead to significant release of BDNF in plasma (Elfving et al., 2010). Therefore, standardized protocols for blood collection, processing, and testing are crucial for improving the accuracy of data.

### 4.7 Effects of different stages of diabetes on BDNF levels in middle-aged and older adults with T2DM

Diabetic patients typically exhibit significant pathophysiological changes, including impaired pancreatic β-cell function, exacerbated insulin resistance, and polyneuropathy (LeRoith, 2002; Eckel et al., 2005). These metabolic disorders may influence BDNF biosynthesis and secretion through multiple mechanisms. Among these, insulin resistance, a core feature of T2DM, has been shown to be significantly associated with circulating BDNF levels (Duan et al., 2003). A study suggests that BDNF may partially compensate for hyperinsulinemia by improving insulin sensitivity, thereby slowing the progression of T2DM (Boyuk et al., 2014). However, some studies have shown that BDNF is negatively correlated with plasma glucose (Krabbe et al., 2007). Especially in the elderly population with T2DM, BDNF secretion function is significantly inhibited. Furthermore, in diabetic populations, reduced serum BDNF levels are significantly associated with impaired glucose metabolism, and this association may further exacerbate the progression of diabetes and its complications (Li et al., 2016). Furthermore, as the duration of T2DM increases, the sustained decrease in BDNF and insulin levels may lead to structural changes in the brain, thereby accelerating cognitive decline in patients (Kandimalla et al., 2017). While exercise can increase BDNF levels in healthy individuals, for T2DM patients, adopting the same exercise regimen as healthy individuals may only alleviate metabolic abnormalities but may not be sufficient to overcome the neurodegnerative changes caused by long-term diabetes. Each middle-aged and elderly T2DM patient has significant differences in disease severity, living environment, and dietary structure, all of which may influence BDNF expression levels. Therefore, for middle-aged and elderly T2DM patients, a multidimensional, personalized exercise intervention strategy should be adopted.

## 5 Limitations

This study followed the PRISMA guidelines and completed PROSPERO registration, utilizing six databases to enhance the reliability of the research findings on the effects of exercise on BDNF concentrations in elderly patients with T2DM. However, this study still has some limitations. First, the number of studies included in this research is limited, and the small sample sizes in some of the included studies may affect the accuracy of the results. Second, exercise effects on BDNF levels in middle-aged and older adults with T2DM may be influenced by factors such as blood collection methods, sampling time, specifications of ELISA kits produced in different countries, age, duration of diabetes, and overall health status. In some trials, all samples were female, which may affect the representativeness of the results and introduce publication bias. Third, in the quality assessment section, some of the included studies had high or unclear risks regarding allocation concealment and blinding type, which increases the likelihood of bias. Fourth, although this study conducted a comprehensive subgroup analysis of exercise parameters (such as frequency, duration, intensity, and mode), there was still high heterogeneity among some subgroup analyses. Fifth, since only one of the included trials involved resistance exercise, the impact of resistance exercise on BDNF levels in middle-aged and older adults with T2DM could not be fully analyzed. Most studies focused on aerobic exercise or combined exercise modes, limiting a comprehensive understanding of the effects of different exercise types. Therefore, future research exploring optimal exercise regimens requires larger sample sizes and higher-quality randomized controlled trials to elucidate the role of potential regulatory factors of BDNF in this population. Additionally, future studies should consider including more diverse exercise types and conducting research across different genders, ages, diabetes duration, and physical health statuses to enhance the generalizability and applicability of the results. In terms of measuring BDNF levels, it is also necessary to standardize blood collection times. For example, when investigating the long-term effects of chronic exercise on BDNF levels in elderly individuals with T2DM, non-immediate blood collection methods can be used. Blood sample testing also requires further selection based on the different sources of BDNF production in the study.

## 6 Conclusion

The results presented in this systematic review and meta-analysis indicate that physical exercise can increase BDNF concentrations in patients with T2DM. Chronic exercise with a duration of at least 150 min per week can significantly increase BDNF levels in middle-aged and elderly individuals with T2DM. However, subgroup analyses of exercise modalities in this study showed that neither aerobic exercise alone nor combined training reached statistical significance. Additionally, when serum BDNF levels were measured using a non-immediate blood draw method after exercise, a significant increase in BDNF concentration was observed. However, no significant differences were found between patients with diabetes duration of ≥10 years or <10 years. Therefore, this systematic review and meta-analysis suggests that long-term, structured exercise programs can increase BDNF levels and improve metabolic health in middle-aged and older adults with T2DM. To better understand the effects of physical exercise on BDNF concentrations in older adults, more diverse intervention studies are needed, particularly those examining the effects of different intervention content and dose-response relationships on BDNF concentrations in middle-aged and older adults with T2DM.

## Data Availability

The original contributions presented in the study are included in the article/Supplementary Material, further inquiries can be directed to the corresponding author.
