# Supplementary material for: Effect of exercise on brain-derived neurotrophic factors in middle-aged and older adults with type 2 diabetes mellitus: a systematic review and meta-analysis
Source: Front Physiol. 2025 Aug 26;16:1599980. doi: 10.3389/fphys.2025.1599980 (PMC12417495; doi:10.3389/fphys.2025.1599980)
Supplement: Supplementary file 1 [file Supplementaryfile1.docx]

**SUPPLEMENTAL MATERIAL**

| **Effect of exercise on brain-derived neurotrophic factors in middle-aged and older adults with type 2 diabetes mellitus: A systematic review and meta-analysis** |
| --- |

[Table S1 Search strategy (November 1, 2024) detailed for PubMed 2](#_Toc29709)

[Table S2 Search strategy (November 1, 2024) detailed for Web of Science 2](#_Toc31576)

[Table S3. Search strategy (November 1, 2024) detailed for EBSCOhost 2](#_Toc15761)

[Table S4. Search strategy (November 1, 2024) detailed for Cochrane Central 3](#_Toc15495)

[Table S5. Search strategy (November 1, 2024) detailed for CNKI 3](#_Toc17652)

[Table S6. Search strategy (November 1, 2024) detailed for Embase 3](#_Toc22827)

[Table S7. Excluded studies by reason for exclusion (N = 76) 4](#_Toc7100)

[Table S8 Future research suggestions on the impact of exercise on BDNF levels in middle-aged and elderly individuals with T2DM. 10](#_Toc32602)

[Figure S1 Risk of bias assessment. 12](#_Toc6082)

[Table S9. PRISMA Abstracts checklist 13](#_Toc30006)

[Table S10. PRISMA checklist. 14](#_Toc22406)

**Table S1 Search strategy (November 1, 2024) detailed for PubMed**

| **ID** | **Query** | **Results** |
| --- | --- | --- |
| 1 | (((((((((((exercise) OR (sport)) OR (aerobic)) OR (exercise intervention)) OR (exercise training)) OR (physical activity)) OR (physical Therapy)) OR (Resistance Training)) OR (aerobic exercise)) OR (Strength Training)) OR (endurance exercise)) OR (functional training) | 1,980,681 |
| 2 | ((((Type 2 Diabetes Mellitus) OR (type 2 diabetes)) OR (diabetes)) OR (T2DM)) OR (diabetes mellitus) | 1,014,724 |
| 3 | ((neurotrophic factor) OR (brain-derived neurotrophic factor)) OR (BDNF) | 87,980 |
| 4 | (((((((((((((exercise) OR (sport)) OR (aerobic)) OR (exercise intervention)) OR (exercise training)) OR (physical activity)) OR (physical Therapy)) OR (Resistance Training)) OR (aerobic exercise)) OR (Strength Training)) OR (endurance exercise)) OR (functional training)) AND (((((Type 2 Diabetes Mellitus) OR (type 2 diabetes)) OR (diabetes)) OR (T2DM)) OR (diabetes mellitus))) AND (((neurotrophic factor) OR (brain-derived neurotrophic factor)) OR (BDNF)) | 366 |

**Table S2 Search strategy (November 1, 2024) detailed for Web of Science**

| **Search** | **Results** |
| --- | --- |
| "(((((((((((ALL=(exercise)) OR ALL=(sport)) OR ALL=(aerobic)) OR ALL=(exercise intervention)) OR ALL=(exercise training)) OR ALL=(physical activity)) OR ALL=(physical Therapy)) OR ALL=(Resistance Training)) OR ALL=(aerobic exercise)) OR ALL=(Strength Training)) OR ALL=(endurance exercise)) OR ALL=(functional training) " | 1378044 |
| "((((ALL=(Type 2 Diabetes Mellitus)) OR ALL=(type 2 diabetes)) OR ALL=(diabetes)) OR ALL=(T2DM)) OR ALL=(diabetes mellitus)" | 733026 |
| "((ALL=(neurotrophic factor)) OR ALL=(brain-derived neurotrophic factor)) OR ALL=(BDNF)" | 48212 |
| #1 AND #2 AND #3 | 353 |

**Table S3. Search strategy (November 1, 2024) detailed for EBSCOhost**

| **ID** | **Search** | **Results** |
| --- | --- | --- |
| S1 | exercise OR sport OR aerobic OR exercise intervention OR exercise training OR physical activity OR physical Therapy OR resistance Training OR aerobic exercise OR strength Training OR  endurance exercise OR functional training | 6,832,559 |
| S2 | Type 2 Diabetes Mellitus OR type 2 diabetes OR diabetes OR T2DM OR diabetes mellitus | 1,551,397 |
| S3 | neurotrophic factor OR brain-derived neurotrophic factor OR BDNF | 96,735 |
| S4 | ( exercise OR sport OR aerobic OR exercise intervention OR exercise training OR physical activity OR physical Therapy OR resistance Training OR aerobic exercise OR strength Training OR  endurance exercise OR functional training ) AND ( Type 2 Diabetes Mellitus OR type 2 diabetes OR diabetes OR T2DM OR diabetes mellitus ) AND ( neurotrophic factor OR brain-derived neurotrophic factor OR BDNF ) | 307 |

**Table S4. Search strategy (November 1, 2024) detailed for Cochrane Central**

| **ID** | **Search** | **Results** |
| --- | --- | --- |
| #1 | (exercise):ti,ab,kw OR (sport):ti,ab,kw OR (aerobic):ti,ab,kw OR (exercise intervention):ti,ab,kw OR (exercise training):ti,ab,kw (Word variations have been searched) | 161870 |
| #2 | (physical activity):ti,ab,kw OR (physical therapy):ti,ab,kw OR (resistance training):ti,ab,kw OR (aerobic exercise):ti,ab,kw OR (strength training):ti,ab,kw (Word variations have been searched) | 153274 |
| #3 | (endurance exercise):ti,ab,kw OR (functional training):ti,ab,kw (Word variations have been searched) | 57431 |
| #4 | #1 OR #2 OR #3 | 257048 |
| #5 | (Type 2 Diabetes Mellitus):ti,ab,kw OR (type 2 diabetes):ti,ab,kw OR (diabetes):ti,ab,kw OR (T2DM):ti,ab,kw OR (diabetes mellitus):ti,ab,kw (Word variations have been searched) | 125835 |
| #6 | (neurotrophic factor):ti,ab,kw OR (brain-derived neurotrophic factor):ti,ab,kw OR (BDNF):ti,ab,kw (Word variations have been searched) | 3013 |
| #7 | #4 AND #5 AND #6 | 75 |

**Table S5. Search strategy (November 1, 2024) detailed for CNKI**

| **Search** | **Results** |
| --- | --- |
| (exercise + sport + aerobic + physical activity + resistance training + endurance exercise + functional training) AND (Type 2 Diabetes Mellitus + T2DM + diabetes mellitus) AND (brain-derived neurotrophic factor + BDNF) | 46 |

**Table S6. Search strategy (November 1, 2024) detailed for Embase**

| **ID** | **Search** | **Results** |
| --- | --- | --- |
| #1 | 'exercise'/exp OR exercise OR 'sport'/exp OR sport OR aerobic OR 'exercise intervention' OR (('exercise'/exp OR exercise) AND('intervention'/exp OR intervention)) OR 'exercise training'/exp OR 'exercise training' OR(('exercise'/exp OR exercise) AND ('training'/exp OR training)) OR 'physical activity'/exp OR 'physical activity' OR (physical AND ('activity'/exp OR activity)) OR 'physical therapy'/exp OR 'physical therapy' OR (physical AND ('therapy'/exp OR therapy)) OR 'resistance training'/exp OR 'resistance training' OR (('resistance'/exp OR resistance) AND ('training'/exp OR training)) OR 'aerobic exercise'/exp OR 'aerobic exercise' OR (aerobic AND ('exercise'/exp OR exercise)) OR 'strength training'/exp OR 'strength training' OR (('strength'/exp OR strength) AND ('training'/exp OR training)) OR 'endurance exercise'/exp OR 'endurance exercise' OR (('endurance'/exp OR endurance) AND ('exercise'/exp OR exercise)) OR 'functional training'/exp OR 'functional training' OR (functional AND ('training'/exp OR training)) | 2,347,237 |
| #2 | 'type 2 diabetes mellitus'/exp OR 'type 2 diabetes mellitus' OR (type AND ('2'/exp OR 2) AND ('diabetes'/exp OR diabetes) AND mellitus) OR 'type 2 diabetes'/exp OR 'type 2 diabetes' OR (type AND ('2'/exp OR 2) AND ('diabetes'/exp OR diabetes)) OR 'diabetes'/exp OR diabetes OR 't2dm'/exp OR t2dm OR 'diabetes mellitus'/exp OR 'diabetes mellitus' OR (('diabetes'/exp OR diabetes) AND mellitus) | 1,702,303 |
| #3 | 'neurotrophic factor'/exp OR 'neurotrophic factor' OR (neurotrophic AND factor) OR 'brain-derived neurotrophic factor'/exp OR 'brain-derived neurotrophic factor' OR ('brain derived' AND neurotrophic AND factor) OR 'bdnf'/exp OR bdnf | 115,682 |
| #4 | #1 AND #2 AND #3 | 757 |

**Table S7. Excluded studies by reason for exclusion (N = 76)**

| **No exercise (n = 34)** | |
| --- | --- |
| 1 | Arentoft, A., Sweat, V., Starr, V., Oliver, S., Hassenstab, J., Bruehl, H., et al. (2009). Plasma BDNF is reduced among middle-aged and elderly women with impaired insulin function: evidence of a compensatory mechanism. *Brain Cogn* 71(2)**,** 147-152. doi: 10.1016/j.bandc.2009.04.009. |
| 2 | Bartholomew, C.L., Muhlestein, J.B., May, H.T., Le, V.T., Galenko, O., Garrett, K.D., et al. (2021). Randomized controlled trial of once-per-week intermittent fasting for health improvement: the WONDERFUL trial. *European heart journal open* 1(2)**,** oeab026. doi: 10.1093/ehjopen/oeab026. |
| 3 | Bazyar, H., Moradi, L., Zaman, F., and Zare Javid, A. (2023). The effects of rutin flavonoid supplement on glycemic status, lipid profile, atherogenic index of plasma, brain-derived neurotrophic factor (BDNF), some serum inflammatory, and oxidative stress factors in patients with type 2 diabetes mellitus: A double-blind, placebo-controlled trial. *Phytotherapy Research* 37(1), 271-284. doi: 10.1002/ptr.7611. |
| 4 | Boyuk, B., Degirmencioglu, S., Atalay, H., Guzel, S., Acar, A., Celebi, A., et al. (2014). Relationship between levels of brain-derived neurotrophic factor and metabolic parameters in patients with type 2 diabetes mellitus. *J Diabetes Res* 2014**,** 978143. doi: 10.1155/2014/978143. |
| 5 | Chiang, Y.H., Li, Y.H., Chan, Y.C., Cheng, Y.C., Wu, J., Lin, J.A., et al. (2024). Low brain-derived neurotrophic factor and high vascular cell adhesion molecule-1 levels are associated with chronic kidney disease in patients with type 2 diabetes mellitus. *Front Endocrinol (Lausanne)* 15, 1403717. doi: 10.3389/fendo.2024.1403717. |
| 6 | Demirkılıç, O., Eski, İ., Çiftçi Öztürk, E., Yasun, Ö., Aydın, B., Birkan, C., et al. (2024). Association Between Dipeptidyl Peptidase-4 Inhibitor Use and Cognitive Functions, Brain-Derived Neurotrophic Factor, and Pentraxin-3 Levels in Patients With Type 2 Diabetes. *Cureus* 16(2)**,** e54440. doi: 10.7759/cureus.54440. |
| 7 | Ding, Z., Cao, L., Jin, R., and Li, R. (2023). Exploration of the Shared Genes and Molecular Pathways between Pre-Eclampsia and Type 2 Diabetes Mellitus via Co-Expression Networks Analysis. *Clinical and Experimental Obstetrics and Gynecology* 50(4). doi: 10.31083/j.ceog5004073. |
| 8 | Galindo-Mendez, B., Trevino, J.A., McGlinchey, R., Fortier, C., Lioutas, V., Novak, P., et al. (2020). Memory advancement by intranasal insulin in type 2 diabetes (MemAID) randomized controlled clinical trial: design, methods and rationale. *Contemporary clinical trials* 89**,** 105934. doi: 10.1016/j.cct.2020.105934. |
| 9 | Gaonkar, B., Prabhu, K., Rao, P., Kamat, A., Rao Addoor, K., and Varma, M. (2020). Plasma angiogenesis and oxidative stress markers in patients with diabetic retinopathy. *Biomarkers* 25(5)**,** 397-401. doi: 10.1080/1354750x.2020.1774654. |
| 10 | Giovanni, P., Domenico, A., Andrea, R., Luigi, S., Antonino, L., Luigi, P.A., et al. (2012). Overactive bladder in subjects with diabetes mellitus. Results of an observational investigation based on the overactive bladder questionnaire. *Neurourology and Urodynamics* 31**,** S28-S29. doi: 10.1002/nau.22259. |
| 11 | Golmohammadi, M., Attari, V.E., Salimi, Y., Nachvak, S.M., and Samadi, M. (2024). The effect of MIND diet on sleep status, anxiety, depression, and cardiometabolic indices in obese diabetic women with insomnia: study protocol for a randomized controlled clinical trial {1}. *Trials* 25(1). doi: 10.1186/s13063-024-08486-y. |
| 12 | Jiang, Y., Wang, S., and Liu, X. (2022). Low serum apelin levels are associated with mild cognitive impairment in Type 2 diabetic patients. *BMC Endocr Disord* 22(1)**,** 137. doi: 10.1186/s12902-022-01051-1. |
| 13 | Júdice, P.B., Magalhães, J.P., Hetherington-Rauth, M., Correia, I.R., and Sardinha, L.B. (2021). Sedentary patterns are associated with BDNF in patients with type 2 diabetes mellitus. *European journal of applied physiology* 121(3)**,** 871-879. doi: 10.1007/s00421-020-04568-2. |
| 14 | Karatas, O., Calan, M., Yuksel, A., Chousein, R., Bozkaya, G., Karatas, M., et al. (2023). The level of the neudesin in type-2 diabetic patients and the relationship between the metabolic parameters and carotid intima-media thickness. *Minerva Endocrinology* 48(3)**,** 288-294. doi: 10.23736/S2724-6507.20.03217-4. |
| 15 | Kong, X., Zhang, X., Zhao, Q., He, J., Chen, L., Zhao, Z., et al. (2014). Association of obesity-related genomic loci and type 2 diabetes among Chinese population. *Circulation* 129. |
| 16 | Liu, J., Yang, W., Luo, H., Ma, Y., Zhao, H., and Dan, X. (2021a). Brain-derived neurotrophic factor Val66Met polymorphism is associated with mild cognitive impairment in elderly patients with type 2 diabetes: a case-controlled study. *Aging Clin Exp Res* 33(6), 1659-1666. doi: 10.1007/s40520-020-01687-w. |
| 17 | Liu, T., Canon, M.D., Shen, L., Marples, B.A., Colton, J.P., Lo, W.-J., et al. (2021b). The Influence of the BDNF Val66Met Polymorphism on the Association of Regular Physical Activity With Cognition Among Individuals With Diabetes. *Biological research for nursing* 23(3)**,** 318-330. doi: 10.1177/1099800420966648. |
| 18 | Makrilakis, K., Liatis, S., Tsiakou, A., Stathi, C., Papachristoforou, E., Perrea, D., et al. (2018). Comparison of health-related quality of Life (HRQOL) among patients with pre-diabetes, diabetes and normal glucose tolerance, using the 15D-HRQOL questionnaire in Greece: the DEPLAN study. *Bmc Endocrine Disorders* 18. doi: 10.1186/s12902-018-0261-3. |
| 19 | Matsuda, T., Suzuki, H., Sugano, Y., Suzuki, Y., Yamanaka, D., Araki, R., et al. (2022). Effects of Branched-Chain Amino Acids on Skeletal Muscle, Glycemic Control, and Neuropsychological Performance in Elderly Persons with Type 2 Diabetes Mellitus: An Exploratory Randomized Controlled Trial. *Nutrients* 14(19). doi: 10.3390/nu14193917. |
| 20 | McCaffery, J.M., Papandonatos, G.D., Huggins, G.S., Peter, I., Kahn, S.E., Knowler, W.C., et al. (2013). FTO predicts weight regain in the Look AHEAD clinical trial. *Int J Obes (Lond)* 37(12)**,** 1545-1552. doi: 10.1038/ijo.2013.54. |
| 21 | Miyamoto, T., Iwakura, T., Matsuoka, N., Iwamoto, M., Takenaka, M., Akamatsu, Y., et al. (2018). Impact of prolonged neuromuscular electrical stimulation on metabolic profile and cognition-related blood parameters in type 2 diabetes: A randomized controlled cross-over trial. *Diabetes Research and Clinical Practice* 142, 37-45. doi: 10.1016/j.diabres.2018.05.032. |
| 22 | Mohammadi, H., Karimifar, M., Heidari, Z., Zare, M., and Amani, R. (2022). The effects of wheat germ consumption on mental health and brain-derived neurotrophic factor in subjects with type 2 diabetes mellitus: a randomized, double-blind, placebo-controlled trial. *Nutritional Neuroscience* 25(1)**,** 46-53. doi: 10.1080/1028415X.2019.1708032. |
| 23 | Mondal, A., Bose, C., Pramanik, S., Dash, D., Mukherjee, B., Malik, R.A., et al. (2024). Circulating netrin-1 levels are reduced and related to corneal nerve fiber loss in patients with diabetic neuropathy. *J Diabetes Investig* 15(8)**,** 1068-1074. doi: 10.1111/jdi.14197. |
| 24 | Ng, Y.T., Phang, S.C.W., Tan, G.C.J., Ng, E.Y., Botross Henien, N.P., UD, M.P., et al. (2020). The Effects of Tocotrienol-Rich Vitamin E (Tocovid) on Diabetic Neuropathy: A Phase II Randomized Controlled Trial. *Nutrients* 12(5). doi: 10.3390/nu12051522. |
| 25 | Park, S., and Daily, J.W. (2016). BDNF RS6265 polymorphisms are associated with lower risk of type 2 diabetes: Gene-nutrient interactions in the Korean genome and epidemiology study. *FASEB Journal* 30. |
| 26 | Saati, S., Dehghan, P., Azizi-Soleiman, F., and Mobasseri, M. (2023). The effect of bitter almond (Amygdalus communis L. var. Amara) gum as a functional food on metabolic profile, inflammatory markers, and mental health in type 2 diabetes women: a blinded randomized controlled trial protocol. *Trials* 24(1). doi: 10.1186/s13063-023-07085-7. |
| 27 | Shehab, M.J., Al-Zubaidi, M.M., and Mohamed, N.S. (2020). Detection of pigment epithelial derived factor polymorphism-5736C/T using quantitative real time pcr in non-proliferative diabetic retinopathy. *Biochemical and Cellular Archives* 20**,** 4371-4375. |
| 28 | Sonne, M.P., Alibegovic, A.C., Hojbjerre, L., Vaag, A., Stallknecht, B., and Dela, F. (2010). Effect of 10 days of bedrest on metabolic and vascular insulin action: a study in individuals at risk for type 2 diabetes. *Journal of Applied Physiology* 108(4)**,** 830-837. doi: 10.1152/japplphysiol.00545.2009. |
| 29 | Spartano, N.L., Davis-Plourde, K.L., Himali, J.J., Murabito, J.M., Vasan, R.S., Beiser, A.S., et al. (2019). Self-reported physical activity and relations to growth and neurotrophic factors in diabetes mellitus: The framingham offspring study. *Journal of Diabetes Research* 2019. doi: 10.1155/2019/2718465. |
| 30 | Sumbul-Sekerci, B., Sekerci, A., Pasin, O., Durmus, E., and Yuksel-Salduz, Z.I. (2023). Cognition and BDNF levels in prediabetes and diabetes: A mediation analysis of a cross-sectional study. *Frontiers in Endocrinology* 14. doi: 10.3389/fendo.2023.1120127. |
| 31 | Sylvetsky, A.C., Issa, N.T., Chandran, A., Brown, R.J., Alamri, H.J., Aitcheson, G., et al. (2017). Pigment Epithelium-Derived Factor Declines in Response to an Oral Glucose Load and Is Correlated with Vitamin D and BMI but Not Diabetes Status in Children and Young Adults. *Hormone Research in Paediatrics* 87(5)**,** 301-306. doi: 10.1159/000466692. |
| 32 | Tabur, S., Oʇuz, E., Sabuncu, T., Korkmaz, H., and Çelik, H. (2015). The effects of calcium channel blockers on nephropathy and pigment epithelium-derived factor in the treatment of hypertensive patients with type 2 diabetes mellitus. *Clinical and Experimental Hypertension* 37(3)**,** 177-183. doi: 10.3109/10641963.2014.933964. |
| 33 | Zeng, B., Yue, Y., Liu, T., Ahn, H., and Li, C. (2022). The Influence of the BDNF Val66Met Variant on the Association Between Physical Activity/Grip Strength and Depressive Symptoms in Persons With Diabetes. *Clinical nursing research* 31(8)**,** 1462-1471. doi: 10.1177/10547738221119343. |
| 34 | Zhen, Y.F., Zhang, J., Liu, X.Y., Fang, H., Tian, L.B., Zhou, D.H., et al. (2013). Low BDNF is associated with cognitive deficits in patients with type 2 diabetes. *Psychopharmacology* 227(1)**,** 93-100. doi: 10.1007/s00213-012-2942-3. |
| **No data of interest (n = 9)** | |
| 1 | Almeida, M.L., Crystine da Silva Sobrinho, A., da Silva Rodrigues, G., Prado, L.S., Rodrigues, K.P., Wiggers, E., et al. (2019). ASSOCIATION OF BDNF AND APOE POLYMORPHISMS IN PRACTITIONERS OF COGNITIVE TRAINING ALLIED TO PHYSICAL ACTIVITY IN AN INLAND TOWN OF BRAZIL. *Alzheimer's and Dementia* 15(7)**,** P1204. doi: 10.1016/j.jalz.2019.06.3633. |
| 2 | Garneau, L., Mulvihill, E.E., Smith, S.R., Sparks, L.M., and Aguer, C. (2024). Myokine Secretion following an Aerobic Exercise Intervention in Individuals with Type 2 Diabetes with or without Exercise Resistance. *International Journal of Molecular Sciences* 25(9). doi: 10.3390/ijms25094889. |
| 3 | Gerbaix, M., Courteix, D., Lac, G., Lesourd, B., Chapier, R., Vinet, A., et al. (2023). A DIETARY PLUS EXERCISE INTERVENTION IMPROVES BONE TURNOVER IN PATIENTS WITH METABOLIC SYNDROME AND TYPE 2 DIABETES. *Aging Clinical and Experimental Research* 35**,** S71. doi: 10.1007/s40520-023-02442-7. |
| 4 | Gholami, F., Khaki, R., Mirzaei, B., and Howatson, G. (2021). Resistance training improves nerve conduction and arterial stiffness in older adults with diabetic distal symmetrical polyneuropathy: A randomized controlled trial. *Experimental Gerontology* 153. doi: 10.1016/j.exger.2021.111481. |
| 5 | Lee, H.H., McGeary, J.E., Dunsiger, S., Baker, L., Balasubramanyam, A., Knowler, W.C., et al. (2021). The Moderating Effects of Genetic Variations on Changes in Physical Activity Level and Cardiorespiratory Fitness in Response to a Life-Style Intervention: A Randomized Controlled Trial. *Psychosomatic Medicine* 83(5)**,** 440-448. doi: 10.1097/PSY.0000000000000930. |
| 6 | Rabinowitz, Y., Ravona-Springer, R., Heymann, A., Moshier, E., Berman, Y., Schwartz, J., et al. (2023). Physical Activity Is Associated with Slower Cognitive Decline in Older Adults with Type 2 Diabetes. *Jpad-Journal of Prevention of Alzheimers Disease* 10(3)**,** 497-502. doi: 10.14283/jpad.2023.26. |
| 7 | Sleddering, M.A., Markvoort, A.J., Dharuri, H.K., Jeyakar, S., Snel, M., Juhasz, P., et al. (2014). Proteomic analysis in type 2 diabetes patients before and after a very low calorie diet reveals potential disease state and intervention specific biomarkers. *PLoS ONE* 9(11). doi: 10.1371/journal.pone.0112835. |
| 8 | Stomby, A., Otten, J., Ryberg, M., Nyberg, L., Olsson, T., and Boraxbekk, C.J. (2017). A Paleolithic Diet with and without Combined Aerobic and Resistance Exercise Increases Functional Brain Responses and Hippocampal Volume in Subjects with Type 2 Diabetes. *Frontiers in Aging Neuroscience* 9. doi: 10.3389/fnagi.2017.00391. |
| 9 | Yoo, M., Kluding, P.M., and D'Silva, L. (2013). Effect of aerobic exercise intervention on painful diabetic neuropathy. *Clinical and Translational Science* 6(2)**,** 115. doi: 10.1111/cts.12047. |
| **Incomplete data (n = 3)** | |
| 1 | Liu, T., Hettish, L., Lo, W.-J., Gray, M., and Li, C. (2021). FEASibility testing a randomized controlled trial of an exercise program to improve cognition for T2DM patients (the FEAST trial): A study protocol. *Research in nursing & health* 44(5)**,** 746-757. doi: 10.1002/nur.22174. |
| 2 | Swift, D.L., Johannsen, N.M., Myers, V.H., Earnest, C.P., Smits, J.A., Blair, S.N., et al. (2012). The effect of exercise training modality on serum brain derived neurotrophic factor levels in individuals with type 2 diabetes. *PloS one* 7(8)**,** e42785. doi: 10.1371/journal.pone.0042785. |
| 3 | Yu, P., Zhu, Z., He, J., Gao, B., Chen, Q., Wu, Y., et al. (2023). Effects of high-intensity interval training, moderate-intensity continuous training, and guideline-based physical activity on cardiovascular metabolic markers, cognitive and motor function in elderly sedentary patients with type 2 diabetes (HIIT-DM): a protocol for a randomized controlled trial. *Frontiers in aging neuroscience* 15**,** 1211990. doi: 10.3389/fnagi.2023.1211990. |
| **Experimental programme (n = 15)** | |
| 1 | ChiCtr (2021). Based on VBM to investigate the effect of acupuncture-rehabilitation therapy on hippocampal volume and its neuroprotective mechanism in patients with vascular cognitive impairment with T2DM. *<https://trialsearch.who.int/Trial2.aspx?TrialID=ChiCTR2100047803>*. |
| 2 | ChiCtr (2022). Effects of different exercise programs on cognitive and motor function, cardiovascular function and biometabolites in sedentary diabetic patients. *<https://trialsearch.who.int/Trial2.aspx?TrialID=ChiCTR2200061573>*. |
| 3 | fwqmfy, R.B.R. (2024). Effects of physical training in aquatic and land environment on type 2 diabetes control. *<https://trialsearch.who.int/Trial2.aspx?TrialID=RBR-10fwqmfy>*. |
| 4 | Irct20090901002394N (2019). Effect of probiotic supplementation in patients with type 2 diabetes. *<https://trialsearch.who.int/Trial2.aspx?TrialID=IRCT20090901002394N44>*. |
| 5 | Irct20190505043476N (2020). Supplementation and aerobic exercise in type 2 diabetics. *<https://trialsearch.who.int/Trial2.aspx?TrialID=IRCT20190505043476N1>*. |
| 6 | Irct20220112053699N (2022). The Effect of Exercise on Physical and Cognitive Function. *<https://trialsearch.who.int/Trial2.aspx?TrialID=IRCT20220112053699N1>*. |
| 7 | Irct20150205020965N (2024a). The effect of camelina powder and yoga practice on type 2 diabetes. *<https://trialsearch.who.int/Trial2.aspx?TrialID=IRCT20150205020965N12>*. |
| 8 | Irct20150205020965N (2024b). The effect of camelina powder on type 2 diabetes. *<https://trialsearch.who.int/Trial2.aspx?TrialID=IRCT20150205020965N11>*. |
| 9 | jRcts (2019). Effect of empagliflozin for HFpEF with Type2 DM. *<https://trialsearch.who.int/Trial2.aspx?TrialID=JPRN-jRCTs071180091>*. |
| 10 | Nct (2020a). Feasibility Testing an Exercise Program to Improve Cognition for T2DM Patients. *<https://clinicaltrials.gov/show/NCT04590833>*. |
| 11 | Nct (2020b). Using Polar Unite Fitness Watch to Improve Cognition for T2DM Patients. *<https://clinicaltrials.gov/show/NCT04603885>*. |
| 12 | Nct (2021). Exercise in Older Adults at Risk for Type 2 Diabetes. *<https://clinicaltrials.gov/show/NCT05229705>*. |
| 13 | Nct (2024). Comparative Analysis of Cost-effectiveness Between Sulfonylureas and DPP4 Inhibitors in Combination With Metformin in Treatment of Type 2 Diabetic Patients : a Retrospective, Observational Study. *<https://clinicaltrials.gov/ct2/show/NCT06570980>*. |
| 14 | Pactr (2024). Development of Novel Role of Trunk Rehabilitation Exercise, Stimulation, and Combined Interventions on BDNF, Enzymatic Antioxidant, and Improving Trunk Control After Stroke. *<https://trialsearch.who.int/Trial2.aspx?TrialID=PACTR202408592508053>*. |
| 15 | Umin (2020). Effect of combined training and detraining on type 2 diabetic women with depression. *<https://trialsearch.who.int/Trial2.aspx?TrialID=JPRN-UMIN000039519>*. |
| **Review (n = 5)** | |
| 1 | Asrih, M., Wei, S., Nguyen, T.T., Yi, H.S., Ryu, D., and Gariani, K. (2023). Overview of growth differentiation factor 15 in metabolic syndrome. *J Cell Mol Med* 27(9)**,** 1157-1167. doi: 10.1111/jcmm.17725. |
| 2 | Bernal, B.M.G., and Salas, L.F. (2023). Diabetes-induced dementia: Review of histopathological changes on autopsy. *Virchows Archiv* 483**,** S147. doi: 10.1007/s00428-023-03602-w. |
| 3 | Cai, Y.H., Wang, Z., Feng, L.Y., and Ni, G.X. (2022). Effect of Exercise on the Cognitive Function of Older Patients With Type 2 Diabetes Mellitus: A Systematic Review and Meta-Analysis. *Frontiers in Human Neuroscience* 16. doi: 10.3389/fnhum.2022.876935. |
| 4 | Jamali, A., Shahrbanian, S., and Morteza Tayebi, S. (2020). The Effects of Exercise Training on the Brain-Derived Neurotrophic Factor (BDNF) in the Patients with Type 2 Diabetes: A Systematic Review of the Randomized Controlled Trials. *Journal of Diabetes and Metabolic Disorders* 19(1)**,** 633-643. doi: 10.1007/s40200-020-00529-w. |
| 5 | Leung, W.K., Yau, S.-Y., Yang, Y., Kwok, A.W., Wong, E.M., Cheung, J.K., et al. (2024). Effects of exercise interventions on brain-derived neurotrophic factor levels in overweight and obesity: A systematic review and meta-analysis. *Journal of exercise science and fitness* 22(4)**,** 278-287. doi: 10.1016/j.jesf.2024.04.001. |

**Table S8 Future research suggestions on the impact of exercise on BDNF levels in middle-aged and elderly individuals with T2DM.**

| **Study**  **Characteristic** | **Recommendations** |
| --- | --- |
| **Population** | Studies on patients with different durations of diabetes:  The duration of diabetes may be a significant factor influencing BDNF levels. Patients with varying disease durations may exhibit considerable differences in their physiological and metabolic states. Future research could further consider stratifying T2DM patients based on disease duration. This may necessitate adjustments to exercise regimens and research designs for patients with different disease durations. |
| **Outcomes** | Research on the impact of resistance exercise on patients with T2DM:  While combined exercise (including resistance exercise) has been shown to significantly improve BDNF levels in middle-aged and older adults with T2DM, there is limited research on the effects of resistance exercise alone on BDNF levels. Future studies could design targeted resistance exercise interventions to compare the effects of different exercise intensities and frequencies of resistance exercise protocols on BDNF levels in T2DM patients. |
| **Study design** | The small sample sizes in some studies may affect the accuracy and reliability of the results. Future research should consider recruiting more participants to enhance the generalizability of the findings. Further exploration is warranted to investigate the effects of different types of exercise on BDNF levels in middle-aged and older adults with T2DM. Beyond conventional cycling and running, future studies could introduce diverse forms of aerobic exercise, such as swimming, dancing, and Tai Chi, to compare their impact on BDNF levels. Incorporating functional exercise (balance, flexibility, and coordination training) into resistance exercise may improve movement ability in daily life and reduce the risk of falls. |
| **Other recommendations** | The studies included in this research come from multiple countries and regions. However, patients with T2DM from different countries and regions vary in lifestyle, dietary habits, and exercise culture. These differences may affect the effects of exercise on BDNF levels and other health indicators. Future research could conduct cross-cultural studies to compare differences in acceptance, adherence, and effectiveness of exercise among T2DM patients from different cultural backgrounds. |


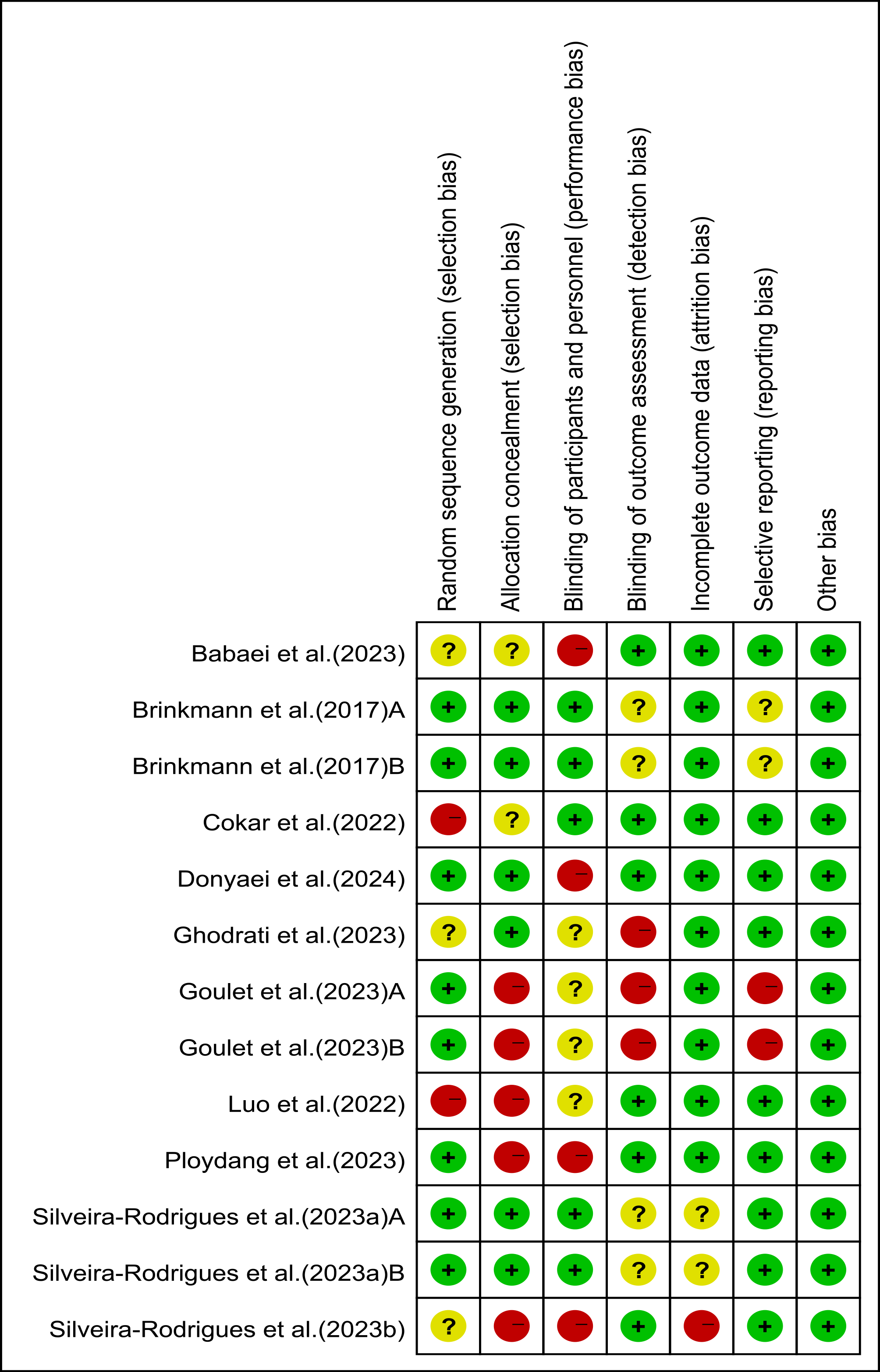


**Figure S1 Risk of bias assessment.**

**Table S9. PRISMA Abstracts checklist**

| **Section and Topic** | **Item #** | **Checklist item** | **Reported (Yes/No)** |
| --- | --- | --- | --- |
| **TITLE** | | |  |
| Title | 1 | Identify the report as a systematic review. | Yes |
| **BACKGROUND** | | |  |
| Objectives | 2 | Provide an explicit statement of the main objective(s) or question(s) the review addresses. | Yes |
| **METHODS** | | |  |
| Eligibility criteria | 3 | Specify the inclusion and exclusion criteria for the review. | Yes |
| Information sources | 4 | Specify the information sources (e.g. databases, registers) used to identify studies and the date when each was last searched. | Yes |
| Risk of bias | 5 | Specify the methods used to assess risk of bias in the included studies. | Yes |
| Synthesis of results | 6 | Specify the methods used to present and synthesise results. | Yes |
| **RESULTS** | | |  |
| Included studies | 7 | Give the total number of included studies and participants and summarise relevant characteristics of studies. | Yes |
| Synthesis of results | 8 | Present results for main outcomes, preferably indicating the number of included studies and participants for each. If meta-analysis was done, report the summary estimate and confidence/credible interval. If comparing groups, indicate the direction of the effect (i.e. which group is favoured). | Yes |
| **DISCUSSION** | | |  |
| Limitations of evidence | 9 | Provide a brief summary of the limitations of the evidence included in the review (e.g. study risk of bias, inconsistency and imprecision). | Yes |
| Interpretation | 10 | Provide a general interpretation of the results and important implications. | Yes |
| **OTHER** | | |  |
| Funding | 11 | Specify the primary source of funding for the review. | No |
| Registration | 12 | Provide the register name and registration number. | Yes |

**Table S10. PRISMA checklist.**

| **Section and Topic** | **Item #** | **Checklist item** | **Location where item is reported** |
| --- | --- | --- | --- |
| **TITLE** | | |  |
| Title | 1 | Identify the report as a systematic review. | P.1 |
| **ABSTRACT** | | |  |
| Abstract | 2 | See the PRISMA 2020 for Abstracts checklist. | P.1 |
| **INTRODUCTION** | | |  |
| Rationale | 3 | Describe the rationale for the review in the context of existing knowledge. | P.2 |
| Objectives | 4 | Provide an explicit statement of the objective(s) or question(s) the review addresses. | P.2 |
| **METHODS** | | |  |
| Eligibility criteria | 5 | Specify the inclusion and exclusion criteria for the review and how studies were grouped for the syntheses. | P.3 |
| Information sources | 6 | Specify all databases, registers, websites, organisations, reference lists and other sources searched or consulted to identify studies. Specify the date when each source was last searched or consulted. | P.2-3 |
| Search strategy | 7 | Present the full search strategies for all databases, registers and websites, including any filters and limits used. | Table S1-S6  (Supplementary material) |
| Selection process | 8 | Specify the methods used to decide whether a study met the inclusion criteria of the review, including how many reviewers screened each record and each report retrieved, whether they worked independently, and if applicable, details of automation tools used in the process. | P.3 |
| Data collection process | 9 | Specify the methods used to collect data from reports, including how many reviewers collected data from each report, whether they worked independently, any processes for obtaining or confirming data from study investigators, and if applicable, details of automation tools used in the process. | P.3 |
| Data items | 10a | List and define all outcomes for which data were sought. Specify whether all results that were compatible with each outcome domain in each study were sought (e.g. for all measures, time points, analyses), and if not, the methods used to decide which results to collect. | P.3 |
|  | 10b | List and define all other variables for which data were sought (e.g. participant and intervention characteristics, funding sources). Describe any assumptions made about any missing or unclear information. | P.3 |
| Study risk of bias assessment | 11 | Specify the methods used to assess risk of bias in the included studies, including details of the tool(s) used, how many reviewers assessed each study and whether they worked independently, and if applicable, details of automation tools used in the process. | P.3 |
| Effect measures | 12 | Specify for each outcome the effect measure(s) (e.g. risk ratio, mean difference) used in the synthesis or presentation of results. | P.3 |
| Synthesis methods | 13a | Describe the processes used to decide which studies were eligible for each synthesis (e.g. tabulating the study intervention characteristics and comparing against the planned groups for each synthesis (item #5)). | P.3 |
|  | 13b | Describe any methods required to prepare the data for presentation or synthesis, such as handling of missing summary statistics, or data conversions. | P.3 |
|  | 13c | Describe any methods used to tabulate or visually display results of individual studies and syntheses. | P.3 |
|  | 13d | Describe any methods used to synthesize results and provide a rationale for the choice(s). If meta-analysis was performed, describe the model(s), method(s) to identify the presence and extent of statistical heterogeneity, and software package(s) used. | P.3 |
|  | 13e | Describe any methods used to explore possible causes of heterogeneity among study results (e.g. subgroup analysis, meta-regression). | P.3 |
|  | 13f | Describe any sensitivity analyses conducted to assess robustness of the synthesized results. | P.3 |
| Reporting bias assessment | 14 | Describe any methods used to assess risk of bias due to missing results in a synthesis (arising from reporting biases). | P.3 |
| Certainty assessment | 15 | Describe any methods used to assess certainty (or confidence) in the body of evidence for an outcome. | P.3 |
| **RESULTS** | | |  |
| Study selection | 16a | Describe the results of the search and selection process, from the number of records identified in the search to the number of studies included in the review, ideally using a flow diagram. | P.4 |
|  | 16b | Cite studies that might appear to meet the inclusion criteria, but which were excluded, and explain why they were excluded. | Table S7 (Supplementary material) |
| Study characteristics | 17 | Cite each included study and present its characteristics. | P.4-5 |
| Risk of bias in studies | 18 | Present assessments of risk of bias for each included study. | P.5 |
| Results of individual studies | 19 | For all outcomes, present, for each study: (a) summary statistics for each group (where appropriate) and (b) an effect estimate and its precision (e.g. confidence/credible interval), ideally using structured tables or plots. | P.5-11 |
| Results of syntheses | 20a | For each synthesis, briefly summarize the characteristics and risk of bias among contributing studies. | P.5 |
|  | 20b | Present results of all statistical syntheses conducted. If meta-analysis was done, present for each the summary estimate and its precision (e.g. confidence/credible interval) and measures of statistical heterogeneity. If comparing groups, describe the direction of the effect. | P.5-11 |
|  | 20c | Present results of all investigations of possible causes of heterogeneity among study results. | P.5-11 |
|  | 20d | Present results of all sensitivity analyses conducted to assess the robustness of the synthesized results. | P.5-11 |
| Reporting biases | 21 | Present assessments of risk of bias due to missing results (arising from reporting biases) for each synthesis assessed. | P.5-11 |
| Certainty of evidence | 22 | Present assessments of certainty (or confidence) in the body of evidence for each outcome assessed. | P.5-11 |
| **DISCUSSION** | | |  |
| Discussion | 23a | Provide a general interpretation of the results in the context of other evidence. | P.13 |
|  | 23b | Discuss any limitations of the evidence included in the review. | P.16-17 |
|  | 23c | Discuss any limitations of the review processes used. | P.16-17 |
|  | 23d | Discuss implications of the results for practice, policy, and future research. | P.16-17 |
| **OTHER INFORMATION** | | |  |
| Registration and protocol | 24a | Provide registration information for the review, including register name and registration number, or state that the review was not registered. | P.2 |
|  | 24b | Indicate where the review protocol can be accessed, or state that a protocol was not prepared. | P.2 |
|  | 24c | Describe and explain any amendments to information provided at registration or in the protocol. | Not applicable |
| Support | 25 | Describe sources of financial or non-financial support for the review, and the role of the funders or sponsors in the review. | Not applicable |
| Competing interests | 26 | Declare any competing interests of review authors. | P.17 |
| Availability of data, code and other materials | 27 | Report which of the following are publicly available and where they can be found: template data collection forms; data extracted from included studies; data used for all analyses; analytic code; any other materials used in the review. | P.17 |
